# Multi-Pass Arrival Time Correction in Cyclic Ion Mobility Mass Spectrometry for Imaging and Shotgun Lipidomics

**DOI:** 10.1021/acsmeasuresciau.4c00077

**Published:** 2024-12-27

**Authors:** Pattipong Wisanpitayakorn, Narumol Jariyasopit, Kassaporn Duangkumpha, Jun Xian Goh, Martin E. Palmer, Yongyut Sirivatanauksorn, Sakda Khoomrung

**Affiliations:** † Siriraj Center of Research Excellence in Metabolomics and Systems Biology (SiCORE-MSB), Faculty of Medicine Siriraj Hospital, Mahidol University, Bangkok 10700, Thailand; ‡ Siriraj Metabolomics and Phenomics Center, Faculty of Medicine Siriraj Hospital, Mahidol University, Bangkok 10700, Thailand; § Thailand Metabolomics Society, Bangkok 10700, Thailand; ∥ Southeast Asia Solution Centre, Waters Pacific Pte Ltd, Singapore 117528, Singapore; ⊥ Waters Corporation, Wilmslow SK9 4AX, U.K.; # Department of Biochemistry, Faculty of Medicine Siriraj Hospital Mahidol University, Bangkok 10700, Thailand; ¶ Center of Excellence for Innovation in Chemistry (PERCH–CIC), Faculty of Science Mahidol University, Bangkok 10400, Thailand

**Keywords:** direct-infusion mass spectrometry, mass spectrometry imaging, cyclic ion mobility mass spectrometry, lipidomics, arrival time correction, shotgun lipidomics

## Abstract

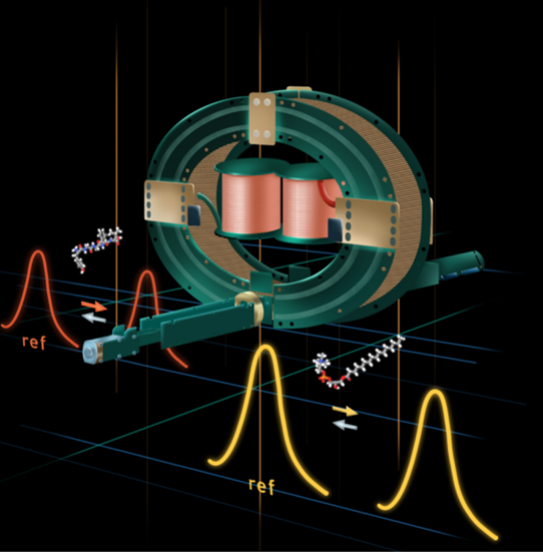

Direct-infusion mass spectrometry (DI-MS) and mass spectrometry imaging (MSI) are powerful techniques for lipidomics research. However, annotating isomeric and isobaric lipids with these methods is challenging due to the absence of chromatographic separation. Recently, cyclic ion mobility mass spectrometry (cIM-MS) has been proposed to overcome this limitation. However, fluctuations in room conditions can affect ion mobility multipass arrival times, potentially reducing annotation confidence. In this study, we developed a multipass arrival time correction method that proved effective across various dates, room temperatures, ion mobility settings, and laboratories using mixtures of reference standards. We observed slight variations in the linear correction lines between lipid and nonlipid molecules, underscoring the importance of choosing appropriate reference molecules. Based on these results, we demonstrated that an accurate multipass arrival time database can be constructed from corrected *t*_0_ and *t*_p_ for interlaboratory use and can effectively identify isomeric lipids in MSI using only a single measurement. This approach significantly simplifies the identification process compared to determining multipass collision cross-section, which requires multiple measurements that are both sample- and time-intensive for MSI. Additionally, we validated our multipass drift time correction method in shotgun lipidomics analyses of human and mouse serum samples and observed no matrix effect for the analysis. Despite variations in dates, room temperatures, instruments, and ion mobility settings, our approach reduced the mean drift time differences from over 2% to below 0.2%.

## 1 Introduction

Direct-infusion mass spectrometry (DI-MS) and mass spectrometry imaging (MSI) are powerful analytical techniques widely used in lipidomics research on various biological samples.^1,2^ Despite their widespread use, DI-MS and MSI lack liquid chromatographic separation, which leads to challenges in distinguishing between isomeric and isobaric species.^1,3−5^ Several studies have coupled ion mobility mass spectrometry (IMS) with DI-MS and MSI to improve lipid identification and annotation.^6−8^ However, to effectively separate and identify ions with very similar geometrical structures, it is crucial to have sufficient ion mobility resolving power.^9^

Cyclic IMS (cIMS) represents a significant advancement in ion mobility technology, offering much higher resolution than traditional low-resolution IMS systems.^10−12^ With a new geometrical design featuring a circular ion mobility chamber with a path length of 98 cm, ions are injected into the chamber and circulate for a prespecified separation time.^10^ After the separation time, the ions that reach the traveling-wave array at the bottom of the cIMS are ejected to the detector. According to previous studies,^13^ the *n*-pass arrival time (*t*_*n*_) of a compound consists of two elements
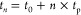
1where *t*_0_ is the zero-pass arrival time, defined as the time taken by the ion to travel through the ion mobility section without completing any passes around the cIMS, and *t*_p_ is the periodic drift time, which corresponds to the time the ions take to complete a cycle through the circular ion mobility device.

Nonetheless, conducting profiling experiments with cIMS poses challenges. First, it is challenging to accurately track the number of passes for each compound. When employing high separation times to enhance separation power, compounds can experience a wrap-around effect, where the faster compounds overtake slower compounds when going around the cyclic ion mobility device.^14^ The second challenge lies in the susceptibility of multipass arrival times to ambient conditions, particularly temperature. To accurately annotate the compound, one can calculate multipass CCS.^15,16^ However, the calculation process increases postanalysis complexity and requires periodic CCS recalibration due to the fluctuation in room conditions. The conversion of traveling wave ion mobility (TWIM) drift time to CCS and the comparison of these values to Drift Tube Ion Mobility (DTIM) measured CCS can introduce further error^15,17^ due to choices of calibrants^18^ and separation principles.^19^ Additionally, the multipass CCS determination requires *t*_p_ measurement. This process demands multiple measurements at various separation times, presenting challenges for mass spectrometry imaging (MSI). In MSI, optimal results are typically achieved by scanning each tissue region only once. Thus, reanalyzing the same region to accurately determine *t*_p_ for all compounds can significantly reduce detection sensitivity due to sample or matrix depletion.^20^ Alternatively, analyzing several different tissue sections consumes a large number of samples, with no guarantee that those sections will capture the same molecular features. Due to the complications associated with multipass CCS, recent multipass cIMS studies of liquid samples of biologically active compounds have primarily focused on resolving selected targeted compounds and comparing their measured multipass arrival times.^21−24^ Furthermore, recent MSI investigations of tissue sections utilized cIMS to annotate the lipids based on the *m*/*z* and MS/MS data without the use of multipass arrival times or multipass CCS.^25,26^

To simplify the compound annotation, a multipass arrival time correction method would enable direct comparison across different experiments and with a database, eliminating the need for multiple measurements or periodic multipass CCS calibration. Previous studies have shown that the drift time measured under slightly different ambient temperature and pressure is correctable, as observed in the drift tube ion mobility spectrometry (DTIMS) studies.^27,28^ LockCCS, a single-point correction with leu-enkephalin, has also been used in TWIM (Synapt G2-Si) to stabilize CCS measurements.^29,30^

In this study, we conducted cyclic ion mobility mass spectrometry (cIM-MS) experiments, utilizing multiple measurements with stepwise increases in separation times, and developed postprocessing pipeline for lipid profiling. We then developed and validated the efficacy of our multipass arrival time correction method using lipid and small molecule standards across various time points, ambient conditions, and instruments. We proposed and demonstrated the use of multipass arrival time database for isomeric lipid annotation using desorption electrospray ionization coupled with cyclic ion mobility mass spectrometry (DESI-cIM-MS). Finally, we validated our method through direct infusion cyclic ion mobility mass spectrometry (DI-cIM-MS) analyses of human and mouse serum samples.

## 2 Experimental Section

### 2.1 Chemicals and Reagents

HPLC-grade acetonitrile, methanol, methyl *tert*-butyl ether (MTBE), and isopropyl alcohol (IPA) were sourced from RCI Labscan and F.N. Science Co., Ltd. (Bangkok, Thailand). Ultrapure water was obtained from a Milli-Q system (Millipore). Leu-enkephalin and the Major Mix calibration solution were from Waters (Milford, USA). Ten lipid standards (>99% purity) were purchased from Avanti Polar Lipids and Sigma-Aldrich (Table S1). The chemical structures of the 10 lipids are illustrated in Figure S1. The standard mixture was prepared by mixing lipid standards with concentrations of 4 μM in ACN/IPA/H_2_O (40:40:20 v/v/v). The lipid standard mixture was also combined with the Major Mix solution (composition listed in Table S2) (1:1 v/v) to investigate the multipass arrival time behavior of compounds from various classes.

### 2.2 Preparation of Human and Mouse Serums

Human serum (product number: H4522) and mouse serum (product number: M5905) were purchased from Sigma-Aldrich. The lipid extraction method used in this study was modified from Matyash et al.^31^ Briefly, 20 μL of serum was mixed with 150 μL of MeOH and vortexed for 30 s. Subsequently, 500 μL of MTBE was added, and the mixture was vortexed at 2000 rpm for 30 min. Following this, 125 μL of Milli-Q water was added, and the solution was vortexed for an additional 30 s. The mixture was incubated at room temperature for 10 min. After the incubation, the solution was centrifuged at 14,000*g* for 15 min at 4 °C. The upper layer containing the lipids was transferred to a new collecting tube. The collected lipids were then dried using a speed vacuum. Once dried, the lipids were reconstituted with 250 μL of MTBE/MeOH (1:1) and vortexed for 1 min. The solution was further sonicated for 15 min at 35 °C. Following sonication, six of these samples were combined into a single LC vial. Finally, lipid standards (LPC 16:0 at 10 μM, LPE 16:0 at 20 μM, PE 16:0/18:1 at 20 μM, and leu-enkephalin at 3.6 μM) were added to the vial as calibrants, resulting in the detection of six adducts (protonated and sodiated for each) in ESI^+^ covering the *m*/*z* range from 454.2934 to 740.5206. The mixture was then sonicated for 10 min before being ready for a direct infusion experiment.

### 2.3 Direct Infusion Cyclic Ion Mobility Mass Spectrometry Analysis

To study the nature of multipass arrival times, a direct infusion experiment was performed on a SELECT SERIES Cyclic IMS (Waters, USA) for the mixture of 4 μM lipid standards in positive (ESI^+^) and negative (ESI^–^) ionization modes. We used the following cyclic ion mobility mass spectrometry (cIM-MS) settings: 3.0 kV capillary voltage, 40 V cone voltage, 100 °C source temperature, 250 °C desolvation temperature, 0 L/h cone gas, 800 L/h desolvation gas, 6.0 bar nebulizer gas, 3.0 kV reference capillary, 10 μL/min infusion flow rate, 6 V trap CE, 4 V transfer CE, and 200 V transfer RF. The high definition MS (HDMS) acquisition mode was used with a *m*/*z* range of 50–1200 Da with a scan time of 1 scan/s. The following cyclic ion mobility parameters were used: 2 pushes per bin, 22 V static traveling wave (TW) height, 375 m/s cyclic TW velocity, 375 m/s array TW velocity, 15 V TW ramp start height, 35 V TW ramp end height, 2.5 V/ms ramping rate, 10 ms injection time, and 26.4 ms ejection and acquisition time. The zero-pass arrival times were collected using the separation time of 0.01 ms. To collect the multipass arrival times of consecutive passes for all lipids in the mixture (*m*/*z* 436.2822 to 785.5899), a series of runs were performed using MassLynx (Waters, USA) with varying separation times from 0.01 to 150 ms. The separation times ranged from 1 to 20 ms in increments of 1 ms, from 20 to 40 ms in increments of 2 ms, from 40 to 100 ms in increments of 5 ms, and from 100 to 150 ms in increments of 10 ms.

All the raw files from series of measurements were processed simultaneously using High Definition Imaging (HDI) software (version 1.6) (Waters, USA), applying the following parameters: HDMS experiment type, number of most intense peaks set to 500, low energy of 10 counts, starting *m*/*z* of 50, stopping *m*/*z* of 1200, *m*/*z* window of 0.02 Da, MS resolution of 20,000, start drift/quad of 1 bin, stop drift/quad of 200 bins, drift/quad window of 1 bin, HD min peak width of 2 bins. The lock-mass option was also enabled (556.2771 for ESI^+^ and 554.2615 for ESI^–^ with lock-mass tolerance of 0.25 amu) for autocorrection of *m*/*z*. After the raw files were processed by the HDI software, a text file containing data of the 500 peaks with the highest signal intensities was generated in a folder called “imaging” within the raw file. The data in this text file were further processed using our in-house developed Python scripts for the following tasks: (1) cleaning and collecting the data from all the text files into one folder, (2) converting all the multipass arrival times from bin units to milli-second units, (3) matching *m*/*z* (within a 10 ppm error) of the 500 peaks with reference *m*/*z* of our expected adduct forms of the lipid standards and export the *m*/*z* and multipass arrival times of the matched compounds, and (4) writing the *m*/*z* and arrival times of all the targeted compounds at all separation times from 0.01 to 150 ms in an Excel file. During the *m*/*z* matching process, if two or more peaks had *m*/*z* within 10 ppm of each other, the peak with highest signal intensity was recorded. For the lipid profiling experiment, 500 peaks with the most signal intensities were processed. Then, periodic drift times of the compounds were calculated from the average multipass arrival times of each pass obtained from the output file. As Critch-Doran et al.^16^ suggested that the periodic drift time of other passes can slightly differ from that of the first pass, we obtained the periodic drift times (*t*_p_) as the slopes between the multipass arrival times and the pass numbers using data of at least 5 passes (eq 1).

### 2.4 DESI-cIM-MS Analysis of Dried Lipid Standards

Five lipid standards (LPC(13:0), LPE(16:0), LPC(16:0), Cer(d18:1/17:0), and PC(16:0/18:0)) were each prepared at a concentration of 200 μM in a solvent mixture of ACN/IPA (40:40:20, v/v/v). A 2 μL aliquot of each solution was spotted adjacent to one another on a glass slide and allowed to dry in a desiccator. The dried lipid spots were then analyzed together in one measurement in positive ionization mode using cIM-MS coupled with DESI XS (Waters, USA). The DESI solvent consisted of MeOH (98:2, v/v) with 0.01% formic acid, and 50 pg/μL leu-enkephalin was added for lock-mass correction. DESI-cIM-MS analysis was performed using the following parameters: 0.7 kV capillary voltage, 40 V sampling cone voltage, 150 °C source temperature, 0.07 MPa DESI gas pressure, 2 μL/min solvent flow rate, 300 μm step size, and a 1 s scan time. The same cIMS settings as those used for the DI-cIM-MS analysis were applied. Following the analysis, the raw data were processed using HDI software with the following settings: HDMS experiment type, 3000 most intense peaks, low energy of 10 counts, starting *m*/*z* of 400, stopping *m*/*z* of 800, *m*/*z* window of 0.02 Da, MS resolution of 20,000, start drift/quad of 1 bin, stop drift/quad of 200 bins, drift/quad window of 1 bin, and HD min peak width of 2 bins. The lock-mass option was also enabled (556.2771 with a lock-mass tolerance of 0.25 amu, sample frequency of 5 min, and sample duration of 10 s) for autocorrection of *m*/*z*.

## 3 Results and Discussion

### 3.1 Interday and Intraday Variation of Multipass Arrival Times

**Figure 1:**
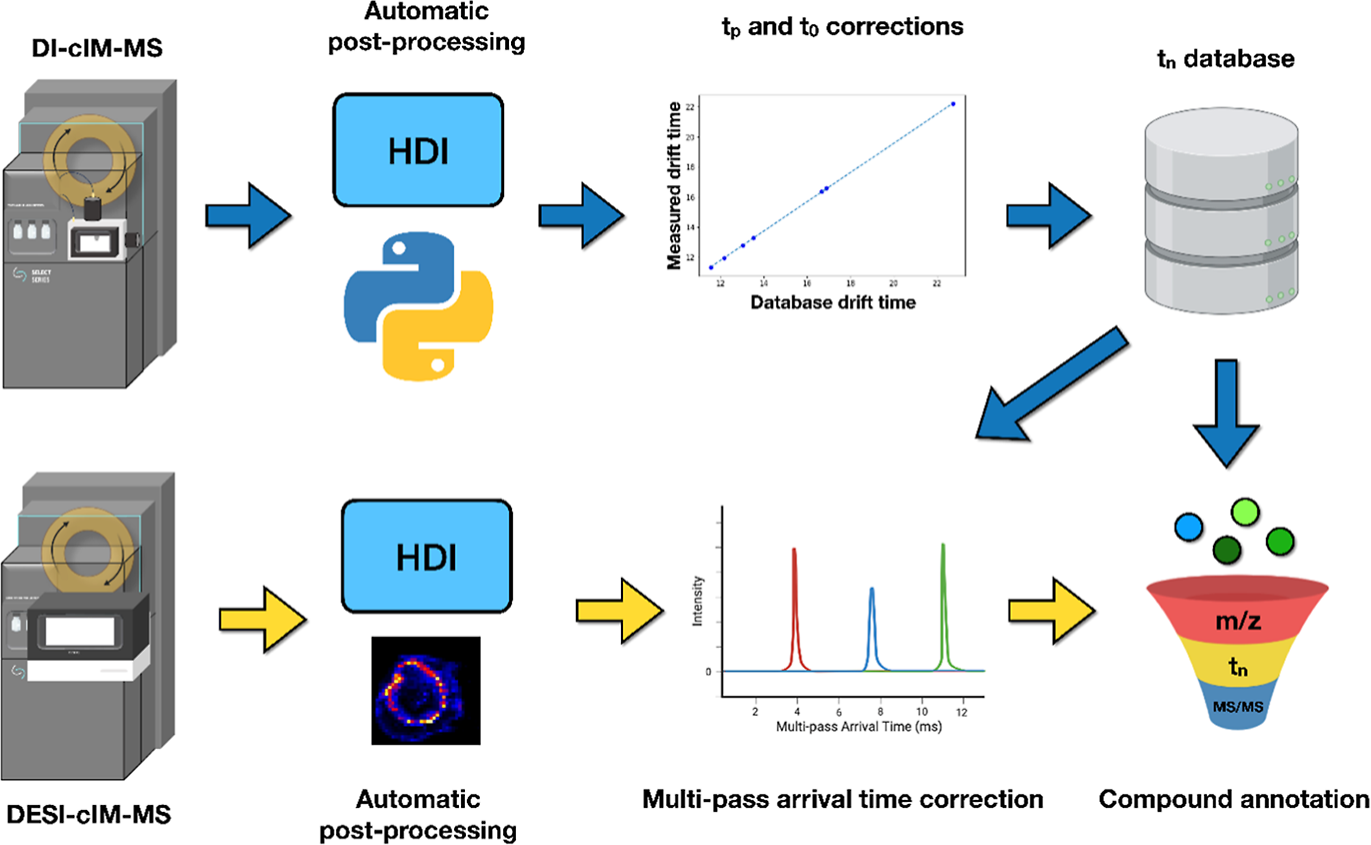
Overview of lipid annotation using our multipass arrival time correction method. A multipass arrival time database can be reconstructed from corrected *t*_0_ and *t*_p_ values measured from a DI-cIM-MS experiment. This database can then be effectively utilized to identify lipids in an MSI experiment based on their *m*/*z*, corrected *t*_n_, and (optional) MS/MS data.

Figure 1 provides an overview of our study. To construct the multipass arrival time database, we assessed the zero-pass arrival times of standard mixtures using a separation time of 0.01 ms. From a single pass experiment, we observed that the single pass drift time of the compounds in this study to be between 5 and 30 ms. Therefore, we measured the multipass arrival times across a specific series of separation times (see Experimental Section for more details). Despite variations in the periodic drift times, which resulted in the wrap-around effect, our measurement scheme enabled us to capture the multipass arrival times of at least the first five consecutive passes for all the compounds in our study. Subsequently, we developed an automated postprocessing pipeline. The HDI software performed automatic peak detection on all raw files simultaneously, while our Python scripts converted the HDI output files into tabulated formats, converted multipass arrival time units from bins to milliseconds for each separation time, and determined *t*_n_ for all passes, *t*_0_, and *t*_p_ of the compounds of interest. Our postanalysis Python script is freely available at https://github.com/MSBSiriraj/Calculating_PeriodicDriftTime under the GNU General Public License (GPL) version 3.

**Figure 2:**
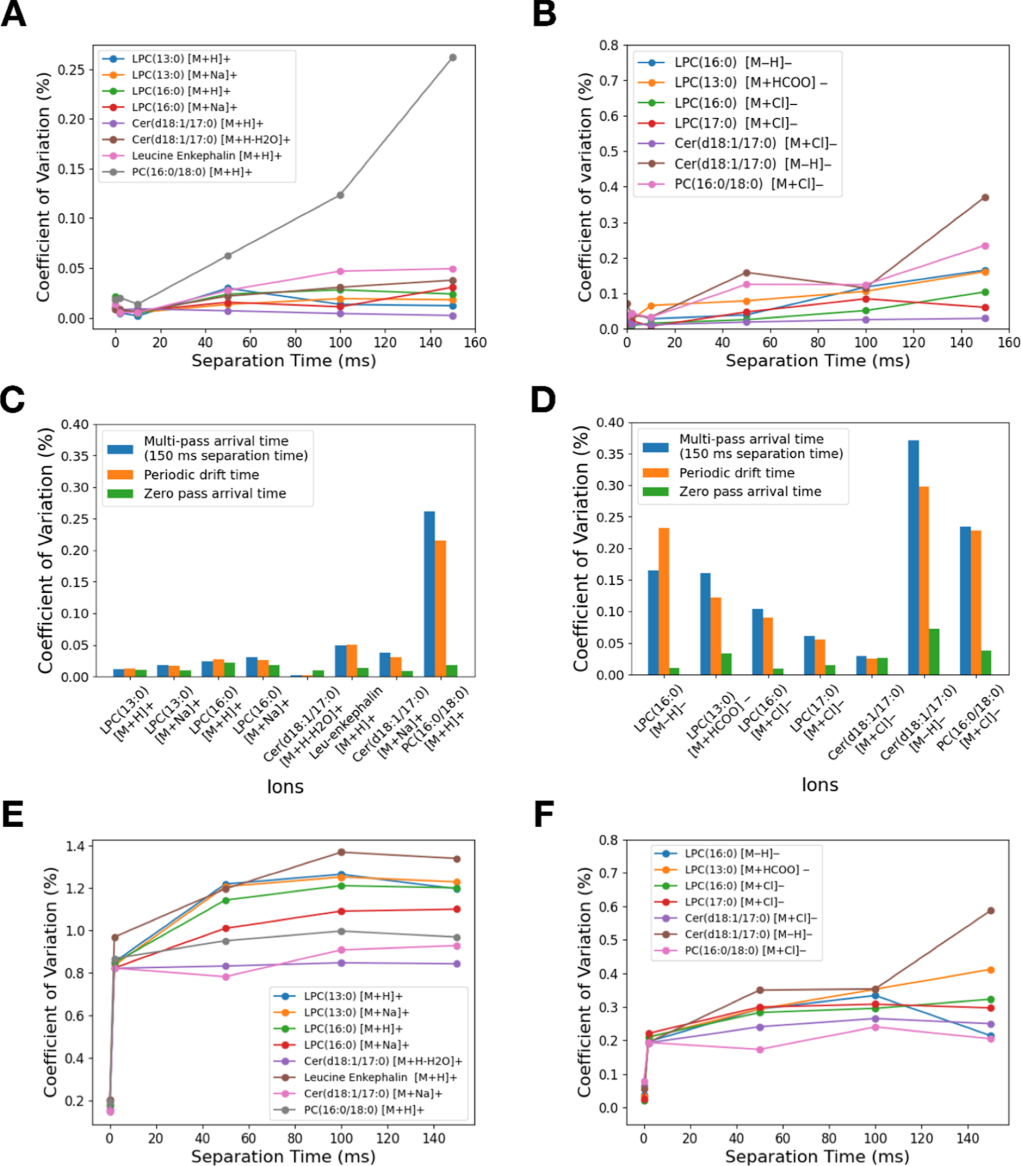
Intraday and interday variation of lipids in cyclic ion mobility spectrometry. (A) Intraday variations of the lipid standards at various separation times in ESI^+^ mode. (B) Intraday variations in ESI^–^ mode. (C) Comparison of intraday variations (*N* = 10) of multipass arrival times at 150 ms separation time, periodic drift times, and zero-pass arrival times in ESI^+^ mode. (D) Same comparison as in (C), but in ESI^–^ mode. (E) Interday variations in ESI^+^ mode. (F) Interday variations in ESI^–^ mode.

To observe the intraday and interday variations of the cIMS, we performed DI-cIM-MS analysis, as detailed in Supporting Information S1. The results obtained from our analysis showed intraday variations of multipass arrival times of less than 0.1% for ESI^+^ and 0.3% for ESI^–^, indicating highly consistent performance for lipids in both ESI^+^ and ESI^–^ modes (see Figure 2A,B), except for protonated PC (16:0/18:0) in ESI^+^ and deprotonated Cer(d18:1/17:0) in ESI^–^. Upon inspection, the significantly higher intraday variations for PC (16:0/18:0) and Cer(d18:1/17:0) were attributed to slightly inaccurate peak picking by the software, caused by the broadening of the arrival time distributions and reduced signals, which resulted in a less defined peak shape. We also compared intraday variations of periodic drift times (*t*_p_) and zero-pass arrival times (*t*_0_) to those of multipass arrival times at a separation time of 150 ms (the longest assessed in this study). As shown in Figure 2C,D and Tables S3 and S4, *t*_0_ exhibited significantly lower intraday variations compared to *t*_p_ and multipass arrival times, likely due to their very short travel distance. Both *t*_p_ and multipass arrival times showed similar levels of variations. We also observed that intraday variations were generally lower in ESI^+^ compared to ESI^–^.

Despite the small intraday variations, cIMS exhibited higher interday variations of multipass arrival times in both ESI^+^ and ESI^–^ modes (Figure 2E,F), likely due to fluctuations in room conditions as listed in Table S5. We found that the multipass arrival times measured from the same week were highly reproducible, with an average of 0.05% for ESI^+^ and 0.39% for ESI^–^ with a separation time of 100 ms. However, interweek comparison showed higher error with an average of 2.38% for ESI^+^ and 0.70% for ESI^–^ with a separation time of 100 ms. The multipass arrival time shift, though minor based on the previous-generation IMS standard, could impede the full potential of compound annotation with cIMS, an instrument with ultrahigh IM resolving power. To demonstrate the challenge, we observed a multipass arrival time difference of 2.6% between protonated LPC(13:0) measured from the interweek comparison in ESI^+^ mode with a separation time of 100 ms. A multipass measurement with the same separation time of a mixture solution of LPC(13:0) and LPE(16:0), which are isomers, showed the multipass arrival times of their protonated forms (*m*/*z* 454.2928) to be 114.11 and 120.84 ms, respectively (Figure S2). If we hypothetically were to perform a lipid profiling experiment of a biological sample at another time point with the exact same settings and found the multipass arrival time of *m*/*z* 454.2928 to be 117.41 ms, approximately 2.9% different from both the measured multipass arrival times of LPC(13:0) and LPE(16:0), it would be difficult to pinpoint whether that compound is LPC(13:0), LPE(16:0), or something else. In addition, a recent study demonstrated closely similar multipass arrival times of isomeric glycolipids measured by cIMS,^32^ underscoring the importance of multipass arrival time correction to ensure optimal compound annotation with cIMS throughout a lipidomic study.

Although fluctuations in room conditions can hinder compound annotation with cIM-MS, we found that the multipass arrival times from different time points could be easily aligned using a linear correction, similar to previous DTIMS studies.^27,28,33^ For example, interweek comparison (day 13 vs day 4) of multipass arrival times of the lipid standards with a separation time of 100 ms had a mean relative error (MRE) of 1.69% in ESI^+^ mode. After applying a linear correction, the MRE was reduced to 0.22% (Figure S3 and Table S6). The extreme precision achieved with the linear correction can greatly increases the confidence in compound annotation with multipass experiments, underscoring the importance of multipass arrival time correction when comparing data between different experiments or against a database.

### 3.2 Accurate Multipass Drift Time Correction

**Figure 3:**
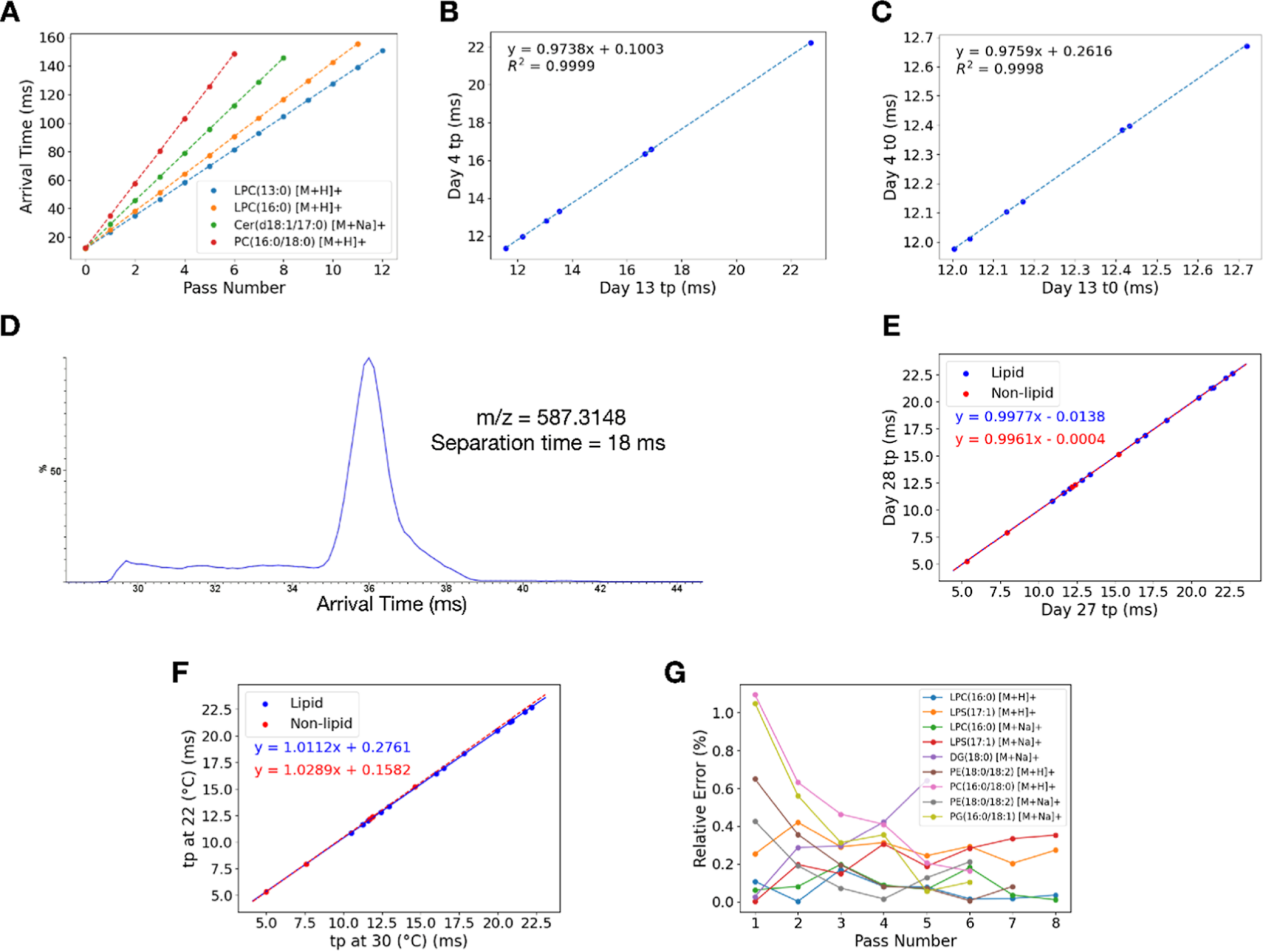
Multipass arrival time correction. (A) Linear relationships observed as a function of multipass arrival time and pass number of various lipid molecules. (B) Linear correction of *t*_p_ from day 13 to day 4. (C) Linear correction of *t*_0_ from day 13 to day 4. (D) Asymmetrically broadened mass extracted multipass arrival time distribution of polyalanine (*m*/*z* = 587.3148) starting to form after 2 passes (separation time = 18 ms). (E) Periodic drift time correction between measurements from 2 different days of a mixture solution containing lipid and nonlipid standards. (F) Periodic drift time correction of a mixture solution containing lipids and nonlipid standards from a measurement at room temperature of 30 °C to that at 22 °C. (G) Relative errors of the reconstructed multipass arrival times of each pass compared to the experimentally measured values.

Despite the correctability of multipass arrival times, when monitoring a specific compound across different experimental time points, variations can occur in the number of passes detected due to fluctuation in the room environments, even when using the same separation time. To address this issue, we conducted the experiments to determine and correct *t*_p_ and *t*_0_. As the multipass arrival times of a compound strictly followed eq 1 (as demonstrated in Figure 3A and previous studies^14,16^), it is possible to determine the corrected multipass arrival times of all passes for each molecule using the corrected *t*_p_ and *t*_0_.

As a result, we observed very strong linear relationships between the *t*_p_ of the compounds in the lipid mixture measured on different weeks, as illustrated by the graph showing ESI^+^ mode *t*_p_ (Figure 3B) and *t*_0_ (Figure 3C) of day 13 vs day 4. As shown in Table S7, using linear fit to correct the interweek *t*_p_ and *t*_0_ significantly reduced the MRE by 12-folds (from 1.97% to 0.16%) for *t*_p_ and 14-fold (from 0.28% to 0.02%) for *t*_0_. Similar trends were also observed in the ESI^–^ mode when correcting the interweek *t*_p_ and *t*_0_, where the MRE of *t*_p_ and *t*_0_ reduced by 13-fold (from 0.79% to 0.06%) and 7-fold (from 0.07% to 0.01%), respectively (Table S8). Therefore, the rest of the study was performed only in ESI^+^ mode, as we expected both ESI^+^ and ESI^–^ modes to yield similar results.

To explore the effectiveness of this correction method across diverse molecular classes, we monitored the multipass arrival times of a mixture containing a broader spectrum of compounds, including 8 lipid standards and 5 nonlipid molecules (benzenoids, peptides, alkaloids) from the Major Mix solution, totaling 23 adducts, in the ESI^+^ mode. We found that polyalanine multipass arrival time distributions (*m*/*z* = 516.2776, 587.3148, 658.3519, 729.3890, etc.) exhibited asymmetrically broadened multipass arrival time distributions after a few passes (Figure 3D), posing challenges for accurate peak picking by the HDI software. Consequently, these compounds were excluded from our analysis. The peaks of verapamil (*m*/*z* = 455.2904) displayed two multipass arrival time peaks with identical signal intensity, as the verapamil protonation introduces a stereocenter.^34^ Although it was possible to correct these peaks, we excluded the verapamil peaks from our analysis due to the difficulty our script would have in distinguishing between the peaks after the wrap-around effect occurred.

We observed that the periodic drift times of small molecules and lipids from the two different days seemed to follow the same linear trend when plotted against each other (Figure 3E). We constructed the correction line using 5 adduct compounds (sulfadimethoxine, Val-Tyr-Val, terfenadine, leu-enkephalin, and reserpine) in the Major Mix solution (Table S9), which was then applied to correct the drift times of the lipids (Table S10). After the correction, we successfully reduced the *t*_p_ MRE of the lipids from 0.32% to 0.08%, although the *t*_0_ MRE increased slightly from 0.06% to 0.10%. However, it is worth noting that the precorrected periodic drift times from these 2 days were not very different, making it difficult to conclude whether lipids and nonlipid molecules shared the same linear correction line.

To further validate the accuracy of using small molecules for correcting the drift times of lipids, we deliberately altered the ambient environment by increasing the room temperature from 22 to 30 °C. The increase in temperature would reduce the periodic drift times of the compounds, primarily due to the heightened kinetic energy of the ions and changes in pressure within the cyclic device. We observed that the periodic drift times for small molecules and lipids formed similar yet slightly different linear lines (Figure 3F). We found that constructing the correction line with small molecules (*R*^2^ > 0.9999) (Table S11) and using it to correct the *t*_p_ and *t*_0_ of the targeted lipids at 30 °C to those at 22 °C resulted in only a 3-fold reduction in MRE from 2.87% to 0.96% for *t*_p_ and 4-fold reduction in MRE from 0.24% to 0.06% for *t*_0_ (Table S12). In contrast, correcting using 5 adducts from 3 lipids (LPE(16:0), Cer(d18:1/17:0), and PC (16:0/18:0)) present in our solution as calibrants (Table S13) yielded highly accurate correction for the rest of the lipids (Table S14), reducing the MRE by 13-fold (from 2.89% to 0.23%) for *t*_p_ and 4-fold (from 0.25% to 0.06%) for *t*_0_. These findings were further confirmed by a replicate experiment, which produced similar results (Tables S15–S18). These results suggested that the linear correction can account for the fluctuation in room conditions. In addition, we found lipids to behave slightly different under the traveling wave compared to other classes of molecules present in the Major Mix, as also observed in CCS calibrations and measurements in the previous generation of traveling wave ion mobility.^18,35−37^ Therefore, it is recommended to perform the correction of lipid multipass arrival time using lipid molecules.

Using eq 1, we also reconstructed the multipass arrival times of these lipids for the first eight passes from the corrected *t*_p_ and *t*_0_ and compared them to the experimentally measured multipass arrival times. It is important to note that the purpose of reconstruction is not to interpolate the data for the higher passes that were not measured but to minimize the error in the values of each pass due to fluctuations in room conditions during the measurements. As shown in Figure 3G, the relative errors of the reconstructed multipass arrival times of the lipids decreased with increasing pass number were below 0.3% for multipass measurements. The only exception was DG(18:0) with increasing *t*_p_. Upon inspection, we observed substantially lower signal intensity for DG(18:0) compared to other lipids. As the pass number increased, the signal intensity of DG(18:0) would decrease, resulting in a less defined peak shape and more error in the peak detection. Nonetheless, our results demonstrated that the corrected *t*_p_ and *t*_0_ could be accurately used to reconstruct multipass arrival times. Based on these results, we believe that the reconstructed multipass arrival time database is invaluable for compound annotation in MSI, where each tissue is typically scanned only once for optimal results. Multipass arrival time and *m*/*z* measured from an MSI experiment can be mapped to the multipass arrival time database to determine which compounds and which pass number this particular feature belongs to.

### 3.3 Isomeric Lipid Annotation Using DESI-cIM-MS with a Multi-Pass Arrival Time Database

In this section, we demonstrated the application of a reconstructed multipass arrival time database by annotating two isomeric compounds, LPC(13:0) and LPE(16:0), using a single DESI-cIM-MS measurement. As our database did not previously contain LPC(13:0), we added its multipass arrival times values to the database by measuring the *t*_p_ and *t*_0_ of a lipid mixture containing LPC(13:0) and three lipids already in the database—LPC(16:0), Cer(d18:1/17:0), and PC(16:0/18:0)—via direct infusion. The measured *t*_p_ values for LPC(13:0) were then corrected through a linear correction lines constructed from the *t*_p_ the protonated and sodiated adducts of the three database lipids as calibrants. Similar correction process was also performed for the measured *t*_0_. The corrected *t*_p_ and *t*_0_ for LPC(13:0), along with the reconstructed multipass arrival times of each pass, were subsequently incorporated into the multipass arrival time database. Table S19 shows the database containing *t*_p_, *t*_0_, and the reconstructed multipass arrival times for the first 10 passes of our lipid standards in ESI^+^.

**Figure 4:**
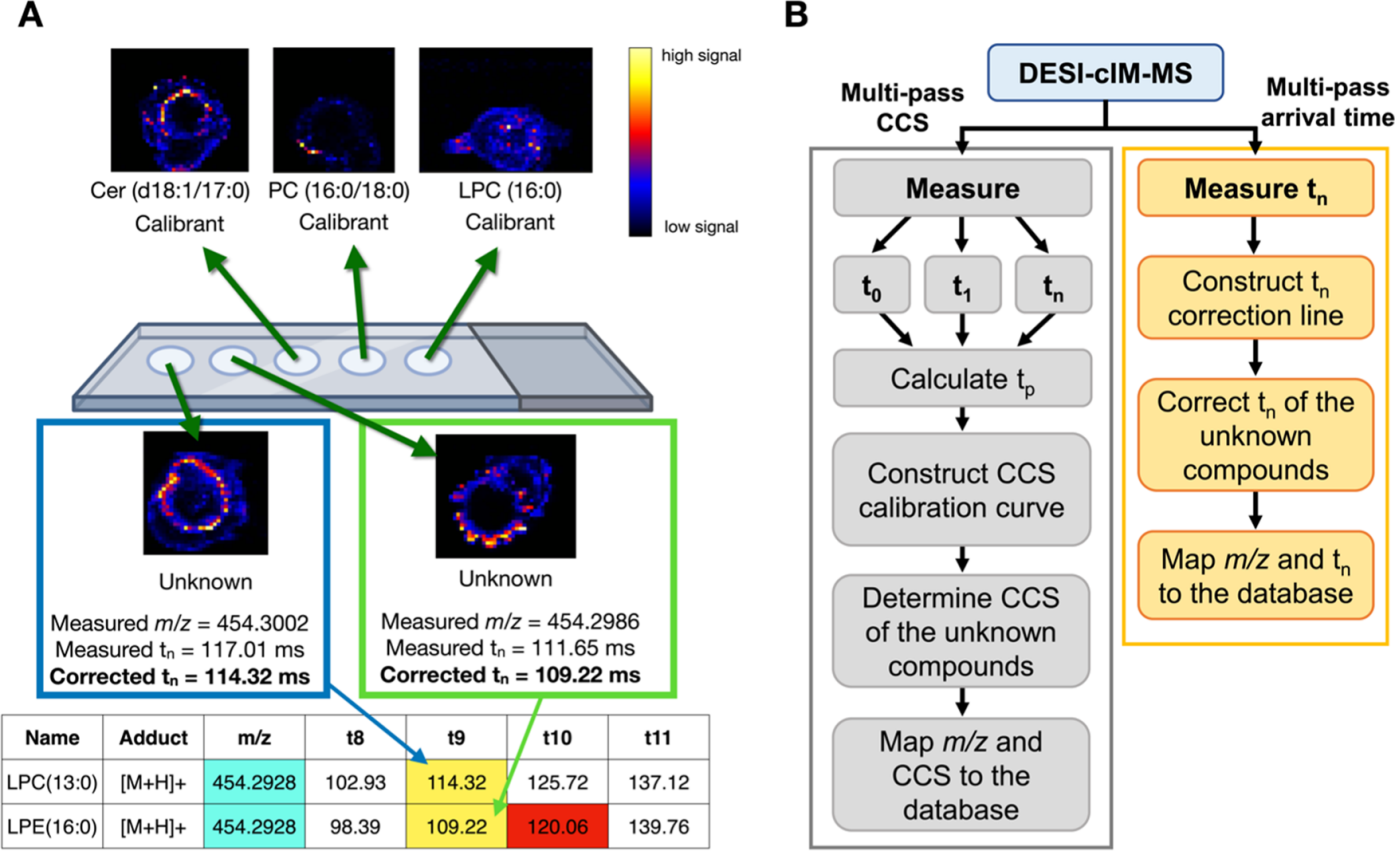
DESI-cIM-MS compound annotation using a multipass arrival time database. (A) Demonstration of isomeric lipid annotation. Five lipid standard spots were analyzed in a single DESI-cIM-MS measurement with a separation time of 100 ms. The corrected multipass arrival times, highlighted in yellow, were directly mapped to the database (Table 20), adding an extra separation dimension beyond *m*/*z* for lipid annotation. Without multipass arrival time correction, the measured *t*_n_ of 117.01 ms could have been incorrectly mapped to pass 10 of LPE(16:0) (highlighted in red). (B) Flowchart illustrating the simplicity of the compound annotation process using corrected multipass arrival time, which requires only a single measurement, compared to multipass CCS, which requires at least three measurements with three different separation times.

Seven months after incorporating LPC(13:0) multipass arrival times into the database, we conducted a DESI-cIM-MS measurement on a glass slide with five dried lipid standard spots, using a separation time of 100 ms, as shown in Figure 4A. LPC(13:0) and LPE(16:0) were the target isomeric lipids, while LPC(16:0), Cer(d18:1/17:0), and PC(16:0/18:0) served as lipid calibrants. Following the analysis, we estimated the number of passes of the measured calibrant lipids by searching the database for the closest *t*_n_ of that particular lipid to the measured *t*_n_ (Table 20). Subsequently, the *t*_n_ correction line was constructed by plotting the measured *t*_n_ values of the protonated and sodiated adducts of the three lipid calibrants against their corresponding database values. To validate our annotation method, we treated two detected features associated with the isomeric lipids (*m*/*z* = 454.3002, *t*_n_ = 117.01 ms and *m*/*z* = 454.2986, *t*_n_ = 111.65) as unknown compounds. Using the correction line, the multipass arrival times of these features were adjusted to 114.32 ms for *m*/*z* 454.3002 and 109.22 ms for *m*/*z* 454.2986. A database search within a 20 ppm *m*/*z* tolerance identified two candidates for these features: LPC(13:0) and LPE(16:0). Further annotation using the corrected multipass arrival times as a second separation dimension revealed that the *t*_n_ values of 114.32 and 109.22 ms were only 0.002% and 0.005% different from the arrival times of pass 9 for LPC(13:0) and LPE(16:0), respectively. This level of accuracy is notable, considering that the precorrected *t*_n_ values differed by 2.35% for LPC(13:0) and 2.22% for LPE(16:0) from the database values. Additionally, without the *t*_n_ correction, the measured *t*_n_ of 117.01 ms could have been mis-annotated as LPE(16:0) pass 10 (*t*_10_ = 120.06 ms).

Our findings demonstrated that the multipass arrival time database and correction are crucial for accurate isomeric lipid annotation in MSI studies. As shown in the flowchart in Figure 4B, our approach, which required only a single measurement, offered more practical compound annotation for MSI studies than using multipass CCS. For multipass CCS determination, at least three separate measurements are required: *t*_0_ (separation time = 0.01 ms), *t*_1_ (separation time ∼2 ms), and *t*_n_ (at the desired separation time). The periodic drift time (*t*_p_) is then calculated using the formula *t*_p_ = (*t*_n_–*t*_0_)/*n*, where *n* = (*t*_n_–*t*_0_)/(*t*_1_–*t*_0_).^13^ A CCS calibration curve can then be constructed with calibrant ions present in the three measurements to convert the measured *t*_p_ to DTIM CCS. Since the process requires three measurements, it can be both time- and sample-consuming for MSI, particularly in cases where each measurement may take days and samples are difficult to obtain, especially from human biopsies. For compound annotation using multipass arrival time, only a *t*_n_ measurement at a desired separation time is needed. The *t*_n_ is then corrected using a calibration curve constructed from calibrant ions applied as a dried standard spot next to the sample. By matching the ion’s *m*/*z* and corrected *t*_n_ to a database, one can not only accurately annotate the ions but also determine the pass number (*n*) for each measured ion. This single-measurement annotation approach can also be applied to rapid shotgun lipidomics analysis, although the multiple-measurement methods of comparing *t*_p_ or multipass CCS may yield slightly more stable values at the cost of increased sample usage, analysis time, and postprocessing complexity.

### 3.4 Validation of Multipass Arrival Time Correction Across Different Instruments

To assess the effectiveness of the correction across different instruments, regardless of ambient conditions, we measured the multipass arrival times of small molecules in the Major Mix solution using two separate instruments: one at the Siriraj Metabolomics and Phenomics Center in Bangkok, Thailand, and the other at Waters Pacific Pte. Ltd. in Singapore. By employing identical experimental methods and postanalysis pipelines as used in the previous Major Mix experiment, we extracted the periodic and zero-pass arrival times of the features corresponding to the five small molecules in the Major Mix solution.

We then constructed the correction lines from the small molecules and used them to align *t*_p_ and *t*_0_ of those molecules from the instrument in Singapore to those obtained from the instrument in Thailand. We found that both *t*_p_ and *t*_0_ obtained from the instruments in Singapore and Thailand have a linear relationship with each other (*R*^2^ > 0.9999), as shown in Figure S4A,B. After correcting the data from Singapore to the data from Thailand using the linear equations, the MRE in *t*_p_ and *t*_0_ reduced significantly from 3.13% to 0.12% and 0.25% to 0.06%, respectively (Table S21). Our results validated that, regardless of variations in room conditions or locations, the multipass arrival times from two cIMS instruments can be accurately aligned using our method. This crucial finding confirms the feasibility of interlaboratory utilization of the multipass arrival time database.

### 3.5 Validation of Multi-Pass Arrival Time Correction in Various Setups for Shotgun Lipid Profiling

**Figure 5:**
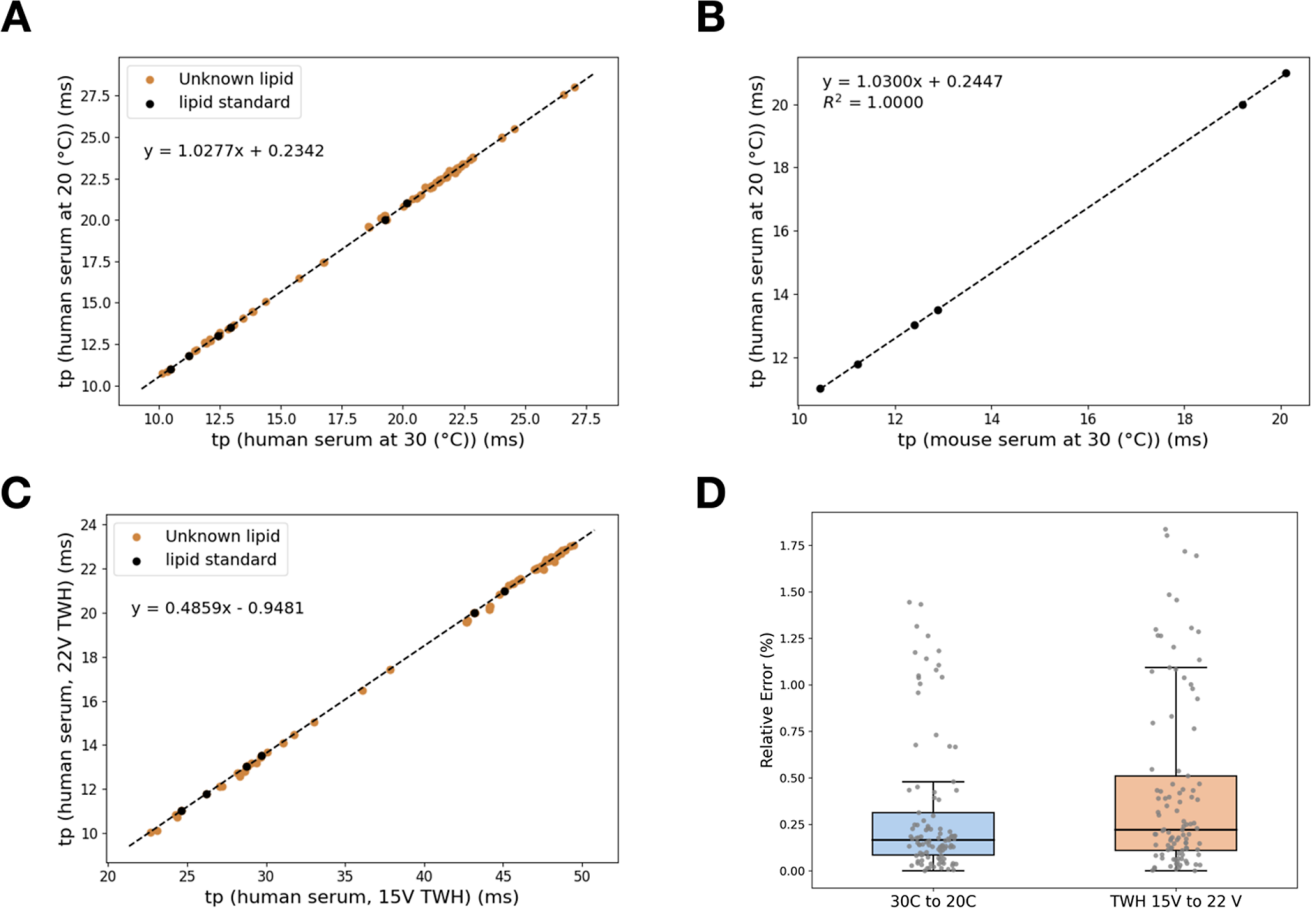
Periodic drift time correction for lipid profiling experiments. (A) Periodic drift time correction of the human serum lipid extract at 20 °C to those at 30 °C. (B) Periodic drift time correction to map the drift time of the spiked lipid standards in mouse serum measured at 30 °C to those in human serum measured at 20 °C. (C) Periodic drift time correction of the human serum lipid extract between a measurement using TWH of 15 V to that of 22 V. The spiked lipid molecules were used to construct the correction lines. (D) Distributions of relative errors in the corrected and experimentally measured *t*_p_ using human serum lipid extract.

Next, we further validated our correction method by applying it to lipid profiling of human and mouse serum samples at room temperatures of 20 and 30 °C. After the automatic postprocessing and manual feature elimination (as discussed in Supporting Information S2 and Figure S5), we initially collected 100 features with the highest signal intensities. We then filtered out nonlipid features by eliminating those whose *m*/*z* values did not match the positive ions listed in the LIPID MAPS Structure Database (LMSD) within a mass tolerance of <0.005 *m*/*z*. Following this screening, 82 features for human serum and 76 features for mouse serum were retained. To evaluate the efficacy of our correction method, we adjusted their *t*_p_ values measured at 30 °C to align with those measured at 20 °C. For both human and mouse serum, we observed a very strong linear relationship for the correction lines (*R*^2^ > 0.9999), based on the spiked lipid molecules (Figures 5A and S6, Tables S22 and S23). As shown in Tables S24 and S25, following correction, the mean relative error (MRE) of *t*_p_ decreased from 4.15% to 0.28% for human serum and from 4.57% to 0.43% for mouse serum.

During the above analysis, we observed that the protonated and sodiated leu-enkephalin, the only nonlipid chemical standard spiked into the serum, displayed high postcorrection errors (>1%), which aligns with our finding regarding to the difference in linear correction trends for lipids and nonlipid molecules. We also found that the majority of the features (72 out of 82 from human serum and 55 out of 76 from mouse serum) displayed small postcorrection error (<0.7%). Based on our previous results with the mixture of lipid and small-molecule standards, we suspected that the features with error >0.7% (highlighted in yellow in Tables S24 and S25) were possibly of nonlipid molecules present in the serum lipid extract that had passed through our initial screening. To validate this hypothesis, we performed a separate self-correction on these high-error features. As anticipated, the correction line for these features differed from that of the low-error features. For the human serum extract, the *t*_p_ MRE of those features reduced from a precorrection value of 5.05% to a postcorrection value of 0.10% instead of 1.14% if corrected with lipid molecules. Similar findings were observed for the mouse serum extract, reducing the *t*_p_ MRE from 5.34% to 0.08% instead of 1.14% if corrected with lipid molecules. Without the nonlipid features, the true reduction in MRE after the linear correction for the lipid features were from 4.02% to 0.16% for the human serum extract and 4.27% to 0.16% for the mouse serum extract. As lipids exhibited a different correction line from nonlipid molecules, this error analysis could provide a viable criterion to screen out nonlipid features in lipidomic analysis.

To investigate matrix effect, we validated the correction across different matrices by correcting the *t*_p_ of the spiked lipid adducts in mouse serum measured at 30 °C to those of the same features in human serum measured at 20 °C. Remarkably, this correction also yielded favorable results across different matrices, reducing the MRE of *t*_p_ from 4.60% to 0.09% (Figure 5B and Table S26). These results showed that the correction performs accurately regardless of the matrices.

In a cyclic ion mobility experiment, traveling wave height (TWH) plays a crucial role in adjusting the velocity of ions and optimizing their separation within the instrument.^15^ Each compound also has its own optimal TWH for maximum signal intensity. For example, we found that the optimal TWH for detecting allulose and fructose, monosaccharides, was 15 V. They could still be detected at 22 V, but not at 30 V or higher (data not shown). Based on this observation, multiple shotgun lipidomics profiling experiments using different TWH may be needed to cover a wide range of compounds. Therefore, we evaluated the performance of our correction by mapping *t*_p_ of the lipid calibrants in human serum obtained at 20 °C under different TWH settings (15 V vs 22 V). Remarkably, we successfully corrected *t*_p_ obtained from the two different TWH settings, resulting in a reduction of MRE from 117.32% to 0.38% (Figure 5C and Tables S27 and S28), respectively. Again, we observed the minority of features with significantly larger errors (>0.7%) than the rest (Figure 5D). Performing a separate correction for the features with large errors resulted in a significantly reduced in MRE, except for the two features (highlighted in red in Table S28) which exhibited significant increase in error from the lipid correction error of 0.76% and 0.83% to the nonlipid correction error of 1.52% and 1.51%, respectively. Thus, these two features were possibly lipid molecules. Upon a close inspection, we found that these two features exhibit nonGaussian multipass arrival time distributions, resulted in inaccurate peak detections and subsequently led to high errors after the drift time correction. By performing separate corrections and excluding the two features, we found that the *t*_p_ MRE reduced from 117.05% to 0.19% for the lipid features, and from 119.40% to 0.36% for the nonlipid features. Thus, in this section, we demonstrated the robustness of our method for multipass arrival time correction across various room conditions, matrix types, and TWH settings, underscoring its versatility and applicability in diverse experimental scenarios.

## 4 Conclusion

This study highlights the effectiveness of multipass arrival time correction in cyclic ion mobility mass spectrometry (cIM-MS), reducing the need for multipass CCS calibration and calculation. We demonstrated the application of our correction method in MSI and shotgun lipidomics to address the absence of liquid chromatographic separation. By utilizing the multipass arrival time database, our method excels in MSI, requiring only a single measurement for accurate lipid annotation, unlike multipass CCS, which necessitates multiple measurements across various separation times. Rigorous experimentation showed that multipass arrival time correction is crucial for cIMS, enhancing the average IM compound annotation confidence to within 0.2% across different time points, ambient conditions, TWH settings, and sample matrices. Since lipids and nonlipid molecules showed slightly different calibration lines, we recommend using calibrants from the same class as the analytes for better accuracy. However, our investigations were only limited to a maximum of 10 passes. Increasing the number of passes, though requiring a more refined separation time series and longer run times, could further increase the confidence of periodic drift times and reconstructed multipass arrival times. Additionally, developing a more advanced postanalysis pipeline capable of handling non-Gaussian multipass arrival time distributions would significantly benefit lipidomic profiling with DI-cIM-MS.

## Supporting Information Available

Supplementary figures and tables containing all the values used in our multipass arrival time corrections (PDF)
