# Supplementary material for: Multi-Pass Arrival Time Correction in Cyclic Ion Mobility Mass Spectrometry for Imaging and Shotgun Lipidomics
Source: ACS Meas Sci Au. 2024 Dec 27;5(1):109–19. doi: 10.1021/acsmeasuresciau.4c00077 (PMC11843504; doi:10.1021/acsmeasuresciau.4c00077)
Supplement: Supplementary file 1 — tg4c00077_si_001.pdf [file tg4c00077_si_001.pdf]

Supplementary Information

Multi-Pass Arrival Time Correction in Cyclic Ion Mobility Mass Spectrometry for Imaging and Shotgun Lipidomics

Pattipong Wisanpitayakorn[1,2,3], Narumol Jariyasopit[1,2,3], Kassaporn Duangkumpha[1,2,3], Jun Xian Goh[4], Martin E. Palmer[5], Yongyut Sirivatanauksorn[2,3,] Sakda Khoomrung[1,2,3,6,7,*]

[1]Siriraj Center of Research Excellence in Metabolomics and Systems Biology (SiCORE-MSB), Faculty of Medicine Siriraj Hospital, Mahidol University, Bangkok 10700, Thailand

[2]Siriraj Metabolomics and Phenomics Center, Faculty of Medicine Siriraj Hospital, Mahidol University, Bangkok 10700, Thailand

[3]Thailand Metabolomics Society, Bangkok, Thailand

[4]Southeast Asia Solution Centre, Waters Pacific Pte Ltd, 117528, Singapore

[5]Waters Corporation, Wilmslow SK9 4AX, United Kingdom

[6]Department of Biochemistry, Faculty of Medicine Siriraj Hospital Mahidol University, Bangkok 10700, Thailand

[7]Center of Excellence for Innovation in Chemistry (PERCH-CIC), Faculty of Science Mahidol University, Bangkok 10400, Thailand

S1. Intraday and Interday Variation Experiments

In a multi-pass experiment, we specified the separation time rather than the number of passes each ion would experience. Thus, to assess the consistency of arrival times in cIM-MS, we examined the intraday (N = 10) and interday (N = 3) variations in multi-pass arrival times for lipid standard mixtures at various separation times, ranging from 0.01 ms (t_0) to 150 ms. A fresh working solution of chemical standards was prepared before each experiment. The temperature and humidity at the beginning of each interday experiment were summarized in **Supplementary Table S2**. It is important to note that the room temperature and humidity were measured by an external thermo-hygrometer and might not accurately represent the actual condition inside the cyclic ion mobility cell. For the interday variation experiment, the first two days were chosen to be within the same week, while the third day was carried out in a different week to observe potential changes in arrival times over time. When assessing the interday variations, the change in ambient conditions might result in a compound experiencing a different number of passes even when measured with the exact same settings, leading to a noticeably different arrival time. In that case, the shifted arrival time was omitted from the analysis.

During the assessment, we observed significantly higher intraday variations for PC(16:0/18:0) in the ESI^+ mode compared to the other compounds. This can be attributed to its multi-pass arrival time peaks exhibiting a slightly broader and flatter top compared to other compounds (**Supplementary Figure S7**). This behavior could be due to its unique ion distribution within the IMS cell or the presence of a second unresolved conformation after seven passes.

S2. Post-processing Details of Shotgun Lipid Profiling

After performing series of measurements with stepwise increases in separation times on human and mouse serums, our automated post-processing pipeline was applied to extract the *m/z*, t_0, and t_p from the lipid extract of human serum at 20°C (as detailed in **Supplementary Information S1**). Using the *m/z* list from the human serum measurement at 20°C, we mapped and obtained the *m/z*, t_0, and t_p of the same features detected in human serum at 30°C and in mouse serum at both 20°C and 30°C. During post-processing, we established that the linear fits in Eq. 1 for all features should yield an R^2 value greater than 0.9990. Additionally, t_0 of the chemical standards used in this study were very narrowly distributed between 11 and 13 ms. The y-intercepts (representing the fitted t_0) significantly divergent from the zero-pass arrival times measured using a separation time of 0.01 ms also acted as an exclusionary criterion. We identified two reasons for removing such features that did not meet these criteria. The first reason was due to the challenge in accurate peak detection of non-Gaussian IM peak shapes. As depicted in **Figure S5A**, a typical peak shape resembles a Gaussian distribution. However, the irregular peak shape in **Figure S5B** typically resulted from a wrap-around effect and reduced signal intensity at higher pass numbers. Peaks depicted in **Figure S5C** typically exhibited a Gaussian distribution at a single pass but deviated from this distribution as the number of passes increased. It is possible that such features have a continuum of conformations or have multiple conformations exist in equilibrium, resulting in constantly changing 'shape'. **Figure S5D** illustrates peaks arising from two or more compounds or conformations with co-eluting arrival times. These features required special attention and manual correction which could not be adequately handled by our automated post-processing pipeline. Therefore, such non-Gaussian peak shapes were excluded from our analysis as they fell outside the scope of this paper, which focuses on validating the multi-pass arrival time correction method. While abovementioned screened out features with non-Gaussian IM peaks, a small

number were still retained. Nevertheless, the retained features generally exhibited higher error rates (>1%) even after the periodic drift time correction compared to features with Gaussian peak shapes. As a result, we manually investigated the features with >1% after-correction errors and eliminated all the features with irregular peak shapes from our analysis.

The second reason for the above criteria was due to the arrival times of certain features could not be detected in some passes. This often occurred with features that initially had low signal intensity in a single pass and lost even more signals as the pass number increased. These skips in passes confused the pass-counting process in our automatic post-processing scripts. Therefore, features with y-intercepts less than 11 ms or more than 13 ms were excluded from our analysis. Furthermore, the measured arrival times of some high passes were skipped for small molecules with t_p lower than 10 ms. This occurred when measuring with a separation time greater than 100 ms in our stepwise measurement, where the separation time was increased by 10 ms at each step. This emphasizes the importance of selecting appropriate series of separation times to cover enough consecutive passes for all compounds of interest to perform a linear fit with confidence. To eliminate these issues in our automatic post-processing, we limited the linear fit analysis to the first eight passes to obtain the t_p, as our experimental scheme was able to consistently capture at least the first eight passes for all small molecules in our study.

Reference

(1) Critch-Doran, O.; Jenkins, K.; Hashemihedeshi, M.; Mommers, A. A.; Green, M. K.; Dorman, F. L.; Jobst, K. J. Toward Part-per-Million Precision in the Determination of an Ion's Collision Cross Section Using Multipass Cyclic Ion Mobility. *Journal of the American Society for Mass Spectrometry* **2024**.

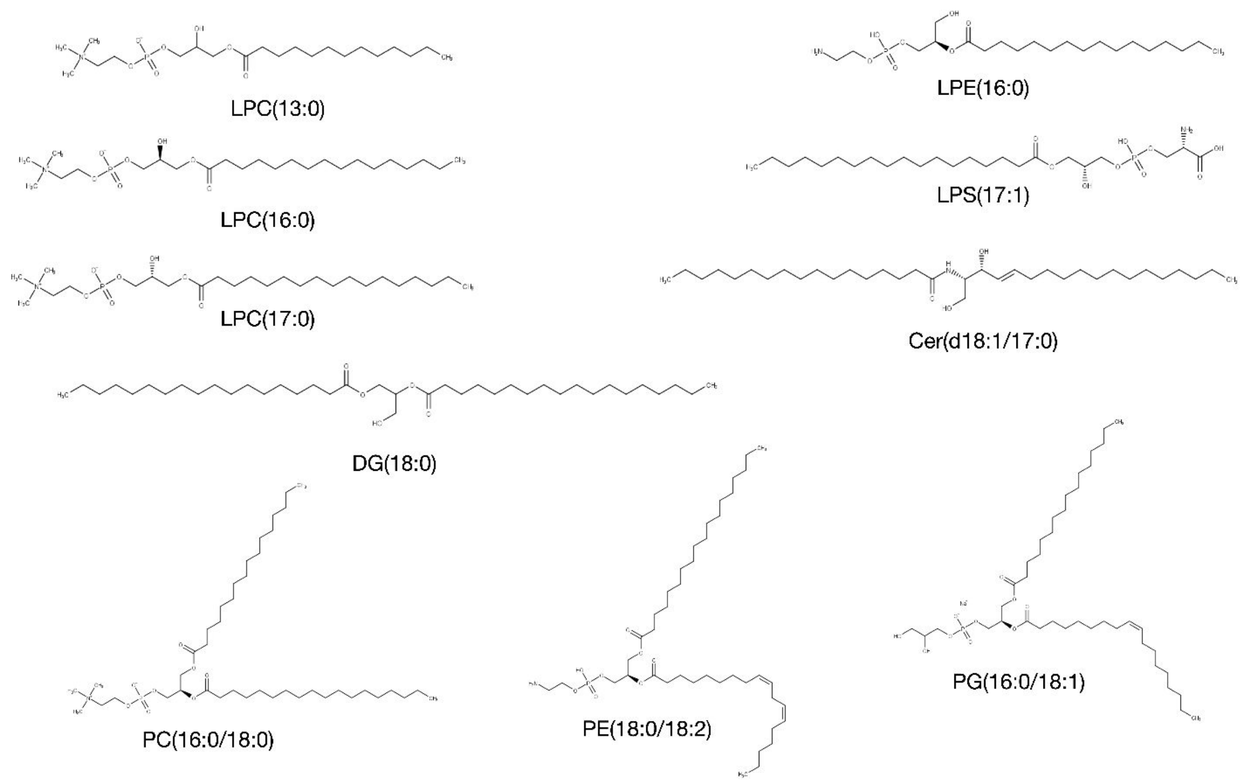

Figure S1: Chemical structures of lipid standards used in this study.

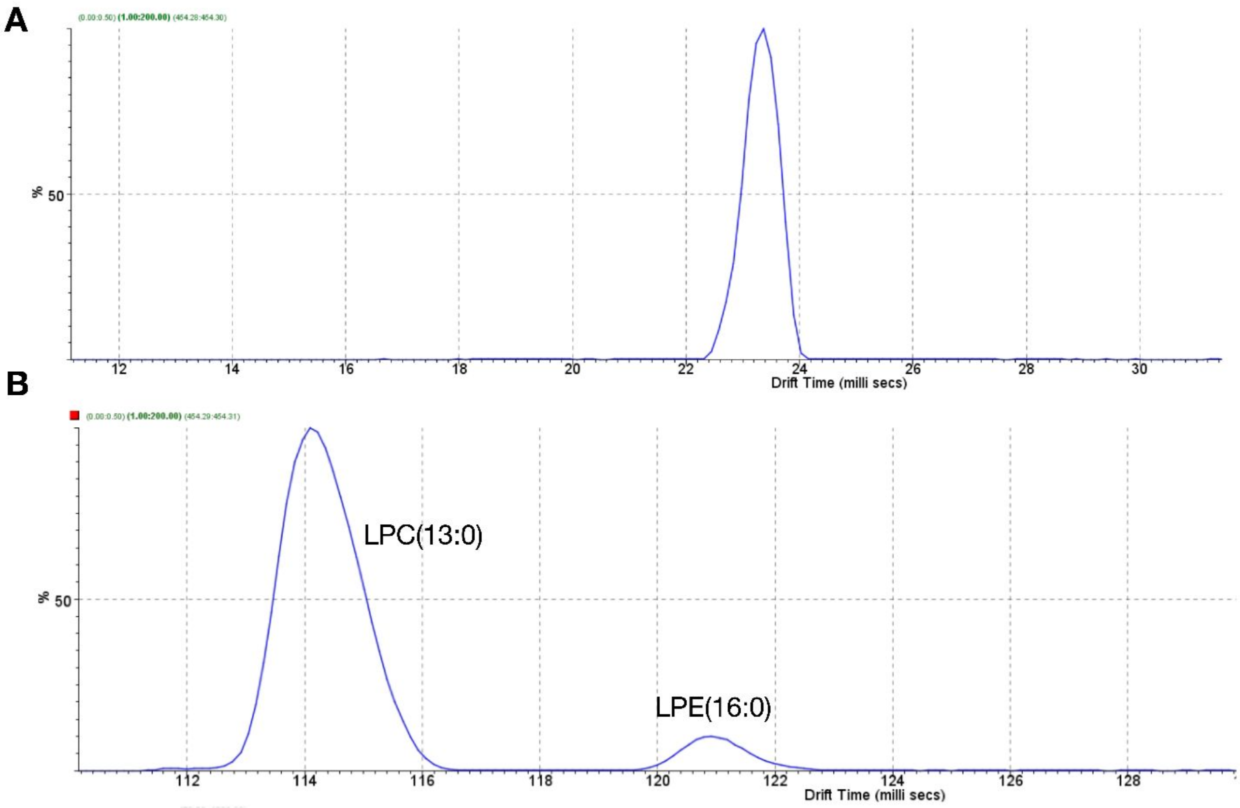

Supplementary Figure S2: Mass extracted multi-pass arrival time peaks (m/z = 454.29 to 454.31) of mixture solution containing LPC(13:0) $[M+H]^+$ and LPE(16:0) $[M+H]^+$ in ESI^+ mode. (A) Co-elution of both isomeric lipids in single-pass experiment with separation time of 2 ms. (B) Clear peak separation of the isomeric lipids in a multi-pass experiment with separation time of 100 ms. The LPC(13:0) and LPE(16:0) were in their 9th and 10th pass, respectively.

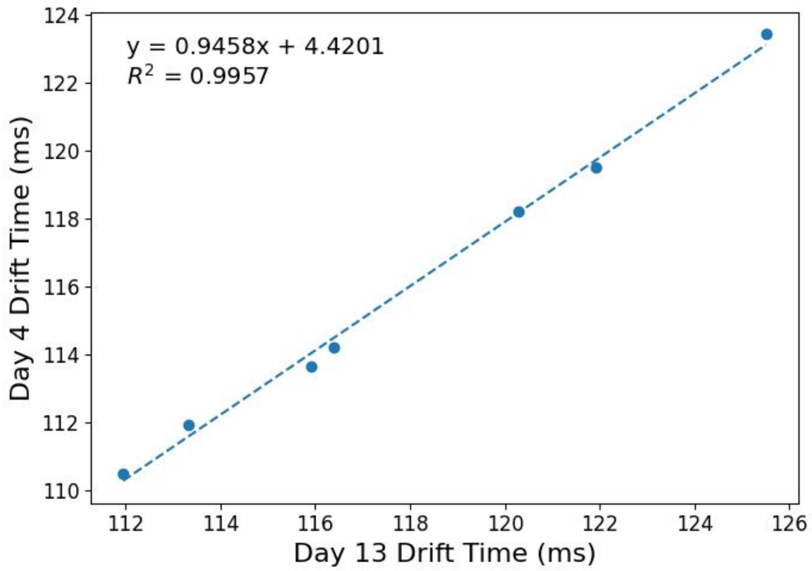

Supplementary Figure S3: Linear correction from the drift times of lipid standards measured on day 13 to those on day 4 (separation time = 100 ms).

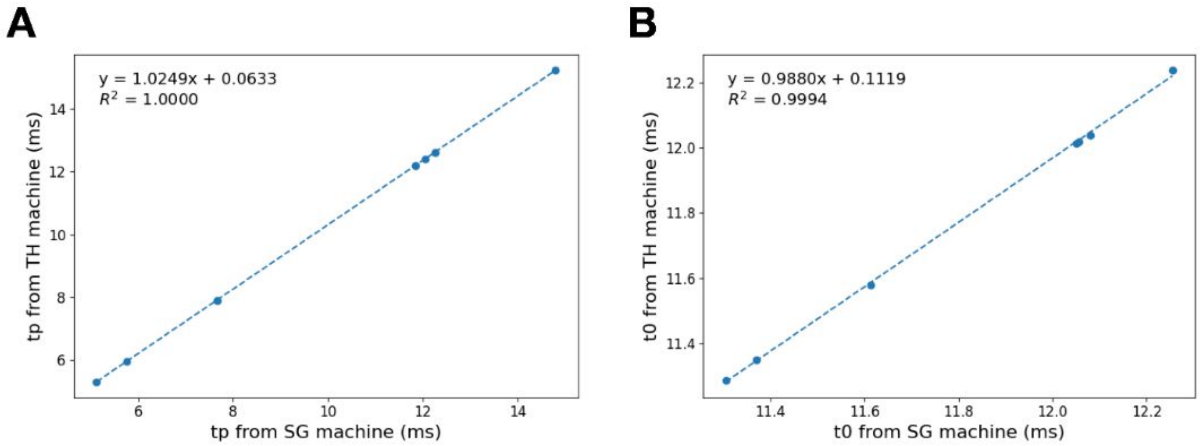

Figure S4: Multi-pass arrival time correction across different instruments. (A) Periodic drift time correction and (B) zero-pass arrival time correction of the Major Mix solution. The corrections were performed to align the drift times obtained from a cIMS in Singapore to those obtained from a cIMS in Thailand.

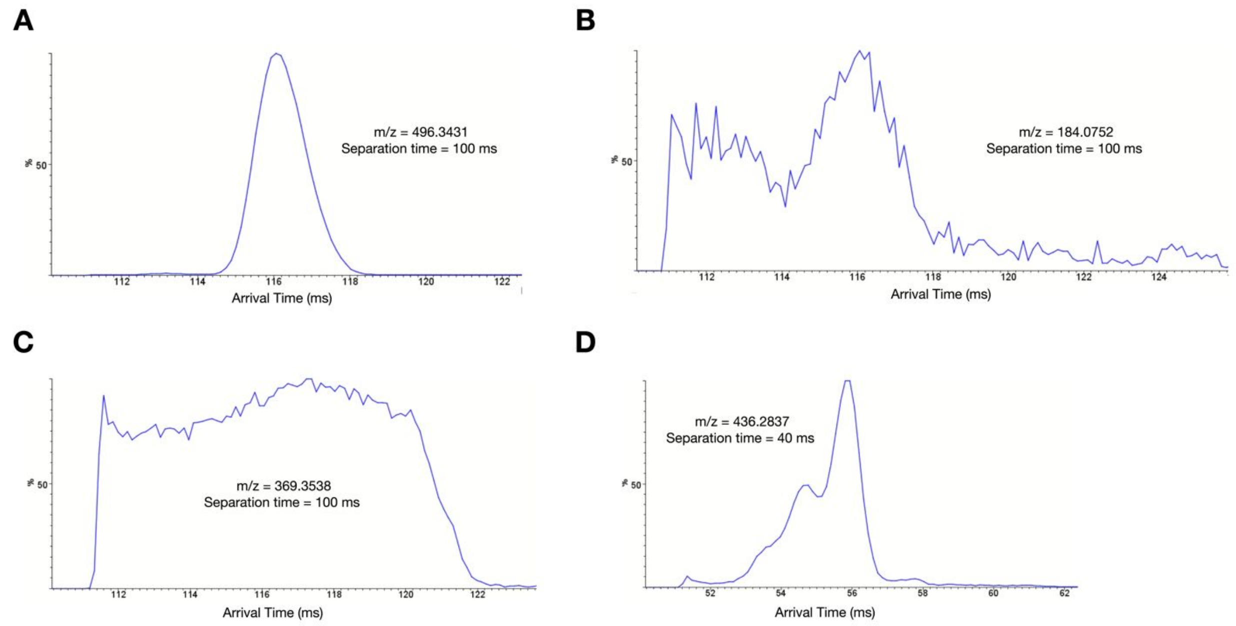

Supplementary Figure S5: Non-gaussian arrival time distributions excluded from this study. (A) Demonstration of a normal gaussian mass extracted arrival time distribution. (B-D) Demonstration of irregular mass extracted arrival time distributions observed in our lipid profiling experiments.

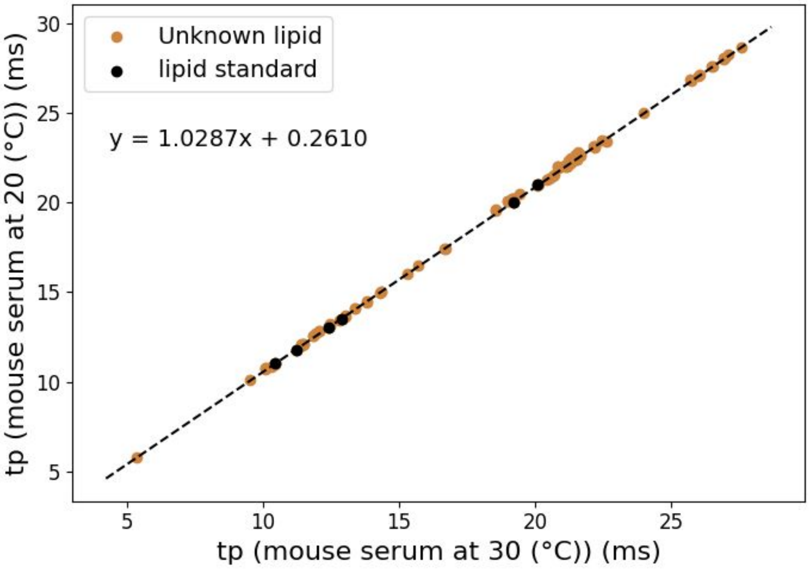

Supplementary Figure S6: Periodic drift time correction of the mouse serum lipid extract at 20°C to those at 30°C.

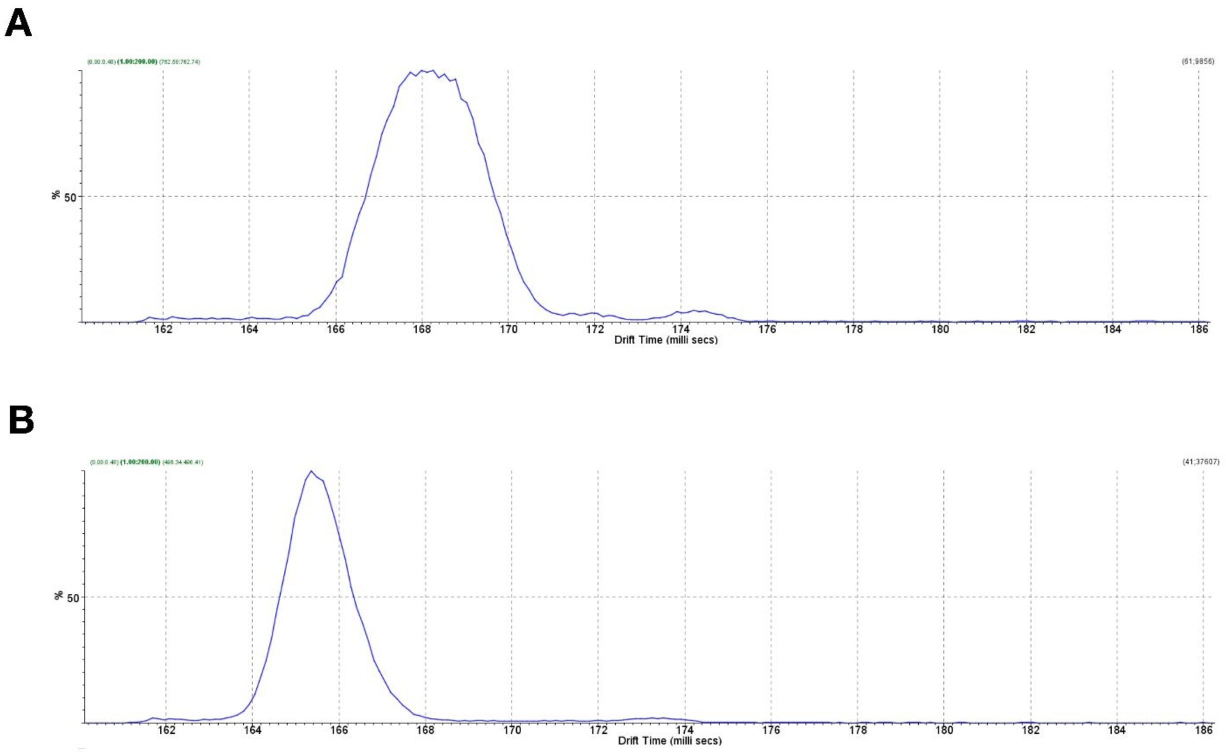

Supplementary Figure S7: Mass extracted multi-pass arrival time peaks of (A) PC(16:0/18:0) $[M+H]^+$ (*m/z* 762.58 to 762.74) and (B) LPC(16:0) $[M+Na]^+$ (*m/z* 496.34 to 496.41) in ESI^+ ionization mode. The measurement was conducted with a separation time of 150 ms.

Supplementary Table S1: List of lipid standards used in this study

No.	Ion mode performed	Exact Mass	CAS No.	Product No.
1	LPC(13:0)	453.2855	20559-17-5	855476P-25MG
2	LPE(16:0)	453.2855	53862-35-4	856705P-200MG
3	LPC(16:0)	495.3325	17364-16-8	855675P-500mg
4	LPC(17:0)	509.3481	50930-23-9	855676P-200MG
5	LPS(17:1)	509.2754	1246298-15-6	858141P-100MG
6	Cer(d18:1/17:0)	551.5277	67492-16-4	860517P-25MG
7	DG(18:0)	624.5693	51063-97-9	800820P-5mg
8	PE(18:0/18:2)	743.5465	7266-53-7	850802C-25MG
9	PG(16:0/18:1)	748.5254	268550-95-4	840457C-500MG
10	PC(16:0/18:0)	761.5935	59403-51-9	850456P-500MG

Supplementary Table S2: List of components in the Major Mix solution. These components are dissolved in approximately 49.5:49.5 Acetonitrile:Water with 0.1% formic acid.

Component	Concentration (µg/mL)
Acetaminophen	0.25
Caffeine	0.0375
Sulfaguanidine	0.125
Sulfadimethoxine	0.025
Val,-Tyr-Val	0.0625
Verapamil	0.005
Terfenadine	0.005
Leucine-enkephalin	0.0625
Reserpine	0.015
Succinic acid	1
Salicylic acid	100
Theophylline	100
Pantothenic acid	10
Stearic acid	200
Perfluoroheptanoic acid	10
Perfluorooctanoic acid	10
Perfluorononanoic acid	10
Perfluorodecanoic acid	10
Perfluorododecanoic acid	10
Poly-DL-alanine	3
Ultramark 1621	1

Supplementary Table S3: Intraday variations (N = 10) of multi-pass arrival time measured with 150 ms separation time, periodic drift time, and zero-pass arrival time of lipids in ESI^+.

Ions	m/z	Coefficient of variation (%)		
		Multi-pass arrival time (150 ms separation time)	Periodic drift time	Zero-pass arrival time
LPC(13:0) $[M+H]^+$	454.2928	0.01	0.01	0.01
LPC(13:0) $[M+Na]^+$	476.2748	0.02	0.02	0.01
LPC(16:0) $[M+H]^+$	496.3398	0.02	0.03	0.02
LPC(16:0) $[M+Na]^+$	518.3217	0.03	0.03	0.02
Cer(d18:1/17:0) $[M+H-H2O]^+$	534.5244	0.00	0.00	0.01
Leu-enkephalin $[M+H]^+$	556.2771	0.05	0.05	0.01
Cer(d18:1/17:0) $[M+Na]^+$	574.517	0.04	0.03	0.01
PC(16:0/18:0) $[M+H]^+$	762.6007	0.26	0.22	0.02
	**AVE**	**0.05**	**0.05**	**0.01**

Supplementary Table S4: Intraday variations (N = 10) of multi-pass arrival time measured with 150 ms separation time, periodic drift time, and zero-pass arrival time of lipids in ESI^-.

Ions	m/z	Coefficient of variation (%)		
		Multi-pass arrival time (150 ms separation time)	Periodic drift time	Zero-pass arrival time
LPC(16:0) $[M–H]^–$	494.3252	0.17	0.23	0.01
LPC(13:0) $[M+HCOO]^–$	498.2837	0.16	0.12	0.03
LPC(16:0) $[M+Cl]^–$	530.3019	0.10	0.09	0.01
LPC(17:0) $[M+Cl]^–$	544.3175	0.06	0.06	0.01
Cer(d18:1/17:0) $[M+Cl]^–$	586.4972	0.03	0.03	0.03
Cer(d18:1/17:0) $[M–H]^–$	550.5205	0.37	0.30	0.07
PC(16:0/18:0) $[M+Cl]^–$	796.5629	0.23	0.23	0.04
	**AVE**	**0.16**	**0.15**	**0.03**

Supplementary Table S5: Room condition at the beginning of each interday variation experiment

Day	Ion mode performed	Room temperature (°C)	Humidity (%)
1	POS and NEG	22.5	58
4	POS and NEG	22.4	55
13	POS	21.8	50
27	NEG	22.2	54

Supplementary Table S6: Demonstration of multi-pass drift time correction from day 13 values to day 4 values. The data were acquired with separation time of 100 ms. The correction equation was y = 0.9458x + 4.4201. Relative errors were used as a benchmark.

Compound	m/z	Pass Number	Day 13 drift time	Day 4 drift time	RE (%)	Drift time day 13 to day 4	Post-correct RE (%)
LPC 13:0 $[M+H]^+$	454.2928	9	115.92	113.66	2.00	114.06	0.36
LPC 13:0 $[M+Na]^+$	476.2748	9	121.92	119.51	2.02	119.73	0.19
LPC 16:0 $[M+H]^+$	496.3398	8	116.40	114.22	1.90	114.51	0.25
LPC 16:0 $[M+Na]^+$	518.3217	8	120.29	118.23	1.74	118.19	0.04
Cer (d18:1/17:0) $[M+H-H2O]^+$	534.5244	6	113.33	111.94	1.24	111.61	0.30
Cer (d18:1/17:0) $[M+Na]^+$	574.517	6	111.95	110.52	1.29	110.30	0.20
PC (16:0/18:0) $[M+H]^+$	762.6007	5	125.50	123.43	1.68	123.12	0.25
				**MRE (%)**	**1.69**		**0.22**

Supplementary Table S7: Corrections of t_p and t_0 of the lipid molecules the positive ion (ESI^+) mode from day 13 values to day 4 values. The correction equations were y = 0.9738x + 0.1003 for t_p and y = 1.0245x - 0.2658 for t_0.

**Compound**	***m/z***	**t_p Day 13 (ms)**	**t_p Day 4 (ms)**	**Pre-correct RE (%)**	**t_p Day 13 to Day 4 (ms)**	**Post-correct RE (%) (ms)**	**t_0 Day 13 (ms)**	**t_0 Day 4 (ms)**	**Pre-correct RE (%)**	**t_0 Day 13 to Day 4 (ms)**	**Post-correct RE (%)**
LPC(13:0) $[M+H]^+$	454.2928	11.57	11.33	2.13	11.37	0.34	12.00	11.98	0.23	11.98	0.00
LPC(13:0) $[M+Na]^+$	476.2748	12.19	11.96	1.88	11.97	0.05	12.04	12.01	0.26	12.01	0.03
LPC(16:0) $[M+H]^+$	496.3398	13.05	12.80	1.95	12.81	0.06	12.13	12.10	0.24	12.10	0.01
LPC(16:0) $[M+Na]^+$	518.3217	13.52	13.29	1.73	13.27	0.18	12.17	12.14	0.28	12.14	0.02
Cer(d18:1/17:0) $[M+H-H_2O]^+$	534.5244	16.89	16.59	1.78	16.55	0.29	12.43	12.40	0.30	12.40	0.01
Cer(d18:1/17:0) $[M+Na]^+$	574.5170	16.67	16.35	1.96	16.34	0.09	12.42	12.38	0.26	12.38	0.05
PC(16:0/18:0) $[M+H]^+$	762.6007	22.73	22.21	2.37	22.24	0.14	12.72	12.67	0.38	12.68	0.03
			**MRE (%)**	**1.97**		**0.16**			**0.28**		**0.02**

Supplementary Table S8: Corrections of tp and t0 of the lipid molecules in the negative ion (ESI$^-$) mode from day 27 values to day 1 values. The correction equations were y = 0.9769x + 0.2384 for t_p and y = 1.0290x - 0.3493 for t_0.

**Compound**	***m/z***	**t_p Day 27 (ms)**	**t_p Day 1 (ms)**	**Pre-correct RE (%)**	**t_p Day 27 to Day 1 (ms)**	**Post-correct RE (%) (ms)**	**t_0 Day 27 (ms)**	**t_0 Day 1 (ms)**	**Pre-correct RE (%)**	**t_0 Day 27 to Day 1 (ms)**	**Post-correct RE (%)**
LPC(16:0) [M-Cl]$^-$	530.3019	13.31	13.24	0.53	13.24	0.01	12.14	12.14	0.01	12.14	0.01
LPC(17:0) [M-Cl]$^-$	544.3175	13.84	13.74	0.68	13.76	0.09	12.17	12.17	0.03	12.17	0.00
Cer (d18:1/17:0) [M-Cl]$^-$	586.4972	15.69	15.59	0.67	15.57	0.13	12.34	12.35	0.07	12.34	0.01
PC (16:0/18:0) [M+Cl]$^-$	796.5629	22.25	21.97	1.27	21.97	0.02	12.70	12.72	0.15	12.72	0.00
			**MRE (%)**	**0.79**		**0.06**			**0.07**		**0.01**

Supplementary Table S9: Corrections from day 28 values to day 27 values of t_p and t_0 of 5 non-lipid molecules in the mixture solution containing Major Mix and lipid standards. The data was used to construct a correction curve, yielding a linear equation of y = 0.9961x - 0.0004 for t_p and y = 1.0108x - 0.1224 for t_0. The post-correction errors shown in this table were calculated based on the self-correction using the two equations.

**Compound**	***m/z***	**t_p Day 28 (ms)**	**t_p Day 27 (ms)**	**Pre-correct RE (%)**	**t_p Day 28 to Day 27 (ms)**	**Post-correct RE (%) (ms)**	**t_0 Day 28 (ms)**	**t_0 Day 27 (ms)**	**Pre-correct RE (%)**	**t_0 Day 28 to Day 27 (ms)**	**Post-correct RE (%)**
Sulfadimethoxine $[M+H]^+$	311.0809	5.30	5.28	0.51	5.28	0.11	11.20	11.20	0.06	11.20	0.05
Val-Tyr-Val $[M+H]^+$	380.2180	7.93	7.91	0.33	7.90	0.07	11.42	11.42	0.01	11.42	0.00
Terfenadine $[M+H]^+$	472.3210	12.19	12.14	0.40	12.14	0.01	11.82	11.84	0.16	11.82	0.11
Leucine Enkephalin $[M+H]^+$	556.2766	12.41	12.37	0.31	12.36	0.08	11.84	11.85	0.09	11.84	0.04
Reserpine $[M+H]^+$	609.2807	15.24	15.17	0.44	15.18	0.04	12.13	12.12	0.02	12.13	0.09
			**MRE (%)**	**0.40**		**0.06**			**0.07**		**0.06**

Supplementary Table S10: Corrections from day 28 values to day 27 values of t_p and t_0 of 8 lipids in the mixture solution containing Major Mix and lipid standards. The tp and t0 corrections in this table were executed based on the correction equations in Supplementary Table S6.

Compound	*m/z*	t_p Day 28 (ms)	t_p Day 27 (ms)	Pre-correct RE (%)	t_p Day 28 to Day 27 (ms)	Post-correct RE (%) (ms)	t_0 Day 28 (ms)	t_0 Day 27 (ms)	Pre-correct RE (%)	t_0 Day 28 to Day 27 (ms)	Post-correct RE (%)
LPE 16:0 $[M+H]^+$	454.2928	10.87	10.83	0.36	10.83	0.04	11.70	11.72	0.17	11.70	0.14
LPE 16:0 $[M+Na]^+$	476.2747	11.65	11.61	0.33	11.61	0.06	11.79	11.81	0.20	11.79	0.16
LPC 16:0 $[M+H]^+$	496.3398	12.83	12.80	0.24	12.78	0.16	11.97	11.97	0.04	11.98	0.01
LPS 17:1 $[M+H]^+$	510.2826	11.64	11.60	0.34	11.59	0.06	11.77	11.79	0.17	11.77	0.13
LPC 16:0 $[M+Na]^+$	518.3216	13.36	13.32	0.33	13.31	0.06	12.04	12.04	0.00	12.05	0.06
LPS 17:1 $[M+Na]^+$	532.2645	12.03	11.98	0.36	11.98	0.03	11.84	11.85	0.11	11.84	0.07
Cer (d18:1/17:0) $[M+H]^+$	552.5350	16.97	16.91	0.38	16.90	0.01	12.35	12.34	0.03	12.36	0.12
Cer (d18:1/17:0) $[M+Na]^+$	574.5169	16.46	16.41	0.31	16.40	0.08	12.29	12.29	0.00	12.31	0.08
DG 18:0 $[M+Na]^+$	647.5584	18.38	18.30	0.44	18.31	0.05	12.41	12.41	0.00	12.42	0.09
PE (18:0/18:2) $[M+H]^+$	744.5538	20.48	20.41	0.36	20.40	0.03	12.53	12.53	0.00	12.54	0.10
PC (16:0/18:0) $[M+H]^+$	763.6080	22.23	22.20	0.15	22.15	0.24	12.72	12.72	0.00	12.73	0.12
PE (18:0/18:2) $[M+Na]^+$	766.5357	21.29	21.26	0.15	21.21	0.24	12.55	12.55	0.02	12.57	0.08

PG 16:0/18:1 [M+Na] $^{+}$	771.5146	21.42	21.34	0.38	21.33	0.01	12.58	12.58	0.01	12.59	0.12
PC (16:0/18:0) [M+Na] $^{+}$	784.5827	22.69	22.62	0.35	22.61	0.05	12.78	12.77	0.03	12.79	0.15
			**MRE (%)**	**0.32**		**0.08**			**0.06**		**0.10**

Supplementary Table S11: Corrections of t_p and t_0 from a measurement at 30°C to map with a measurement at 22°C of 5 non-lipid molecules in the mixture solution containing Major Mix and lipid standards. The data was used to construct a correction curve, yielding a linear equation of $y = 1.0289x + 0.1582$ for t_p and $y = 1.0363x - 0.4087$ for t_0. The post-correction errors shown in this table were calculated based on the self-correction using the two equations.

**Compound**	***m/z***	**t_p 30°C (ms)**	**t_p 22°C (ms)**	**Pre-correct RE (%)**	**t_p 30°C to 22°C (ms)**	**Post-correct RE (%) (ms)**	**t_0 30°C (ms)**	**t_0 22°C (ms)**	**Pre-correct RE (%)**	**t_0 30°C to 22°C (ms)**	**Post-correct RE (%)**
Sulfadimethoxine $[M+H]^+$	311.0809	4.98	5.30	6.13	5.28	0.44	11.19	11.20	0.07	11.19	0.09
Val-Tyr-Val $[M+H]^+$	380.2180	7.58	7.93	4.45	7.96	0.30	11.42	11.42	0.01	11.43	0.06
Terfenadine $[M+H]^+$	472.3210	11.71	12.19	3.93	12.21	0.15	11.81	11.82	0.07	11.83	0.10
Leucine Enkephalin $[M+H]^+$	556.2771	11.91	12.41	4.04	12.41	0.01	11.82	11.84	0.16	11.84	0.02
Reserpine $[M+H]^+$	609.2807	14.64	15.24	3.94	15.22	0.13	12.08	12.13	0.35	12.11	0.10
			**MRE (%)**	**4.50**		**0.21**			**0.13**		**0.07**

Supplementary Table S12: Corrections of t_p and t_0 from a measurement at 30°C to map with a measurement at 22°C of of 8 lipids in the mixture solution containing Major Mix and lipid standards. The t_p and t_0 corrections in this table were executed based on the correction equations in Supplementary Table S8.

Compound	*m/z*	t_p 30°C (ms)	t_p 22°C (ms)	Pre-correct RE (%)	t_p 30°C to 22°C (ms)	Post-correct RE (%) (ms)	t_0 30°C (ms)	t_0 22°C (ms)	Pre-correct RE (%)	t_0 30°C to 22°C (ms)	Post-correct RE (%)
LPE 16:0 $[M+H]^+$	454.2928	10.50	10.87	3.42	10.96	0.83	11.70	11.70	0.02	11.72	0.16
LPE 16:0 $[M+Na]^+$	476.2747	11.27	11.65	3.32	11.75	0.83	11.79	11.79	0.01	11.81	0.15
LPC 16:0 $[M+H]^+$	496.3398	12.43	12.83	3.08	12.95	0.95	11.92	11.94	0.18	11.94	0.02
LPS 17:1 $[M+H]^+$	510.2826	11.22	11.64	3.57	11.70	0.57	11.76	11.77	0.03	11.78	0.12
LPC 16:0 $[M+Na]^+$	518.3216	12.96	13.36	2.98	13.49	1.00	12.01	12.04	0.26	12.04	0.04
LPS 17:1 $[M+Na]^+$	532.2645	11.59	12.03	3.64	12.08	0.46	11.82	11.84	0.10	11.84	0.07
Cer (d18:1/17:0) $[M+H]^+$	552.5350	16.51	16.97	2.70	17.15	1.05	12.29	12.35	0.43	12.33	0.12
Cer (d18:1/17:0) $[M+Na]^+$	574.5169	16.03	16.46	2.61	16.65	1.16	12.26	12.29	0.29	12.30	0.01
DG 18:0 $[M+Na]^+$	647.5584	17.80	18.38	3.16	18.47	0.50	12.37	12.41	0.31	12.41	0.02
PE (18:0/18:2) $[M+H]^+$	744.5538	19.97	20.48	2.47	20.71	1.12	12.49	12.53	0.35	12.53	0.01
PC (16:0/18:0) $[M+H]^+$	763.6080	21.76	22.23	2.13	22.55	1.41	12.67	12.72	0.35	12.73	0.05
PE (18:0/18:2) $[M+Na]^+$	766.5357	20.75	21.29	2.53	21.51	1.03	12.52	12.55	0.26	12.57	0.10

PG 16:0/18:1 [M+Na]$^{+}$	771.5146	20.90	21.42	2.41	21.66	1.15	12.53	12.58	0.38	12.58	0.01
PC (16:0/18:0) [M+Na]$^{+}$	784.5827	22.21	22.69	2.14	23.01	1.38	12.73	12.78	0.40	12.78	0.01
			**MRE (%)**	**2.87**		**0.96**			**0.24**		**0.06**

Supplementary Table S13: Corrections of t_p and t_0 from a measurement at 30°C to map with a measurement at 22°C of 5 lipid adducts from 3 different lipids in the mixture solution containing Major Mix and lipid standards. The lipids were selected based on them having the lowest, medium, and high arrival times to cover the arrival time range of our lipids. The data was used to construct a correction curve, yielding a linear equation of $y = 1.0098x + 0.2765$ for t_p and $y = 1.0595x - 0.6949$ for t_0. The post-correction errors shown in this table were calculated based on the self-correction using the two equations.

**Compound**	***m/z***	**t_p 30°C (ms)**	**t_p 22°C (ms)**	**Pre-correct RE (%)**	**t_p 30°C to 22°C (ms)**	**Post-correct RE (%) (ms)**	**t_0 30°C (ms)**	**t_0 22°C (ms)**	**Pre-correct RE (%)**	**t_0 30°C to 22°C (ms)**	**Post-correct RE (%)**
LPE(16:0) $[M+H]^+$	454.2928	10.50	10.87	3.42	10.88	0.07	11.70	11.70	0.02	11.71	0.04
LPE(16:0) $[M+Na]^+$	476.2747	11.27	11.65	3.32	11.65	0.00	11.79	11.79	0.01	11.79	0.04
Cer(d18:1/17:0) $[M+H]^+$	552.5350	16.51	16.97	2.70	16.95	0.11	12.29	12.35	0.43	12.33	0.13
Cer(d18:1/17:0) $[M+Na]^+$	574.5169	16.03	16.46	2.61	16.47	0.02	12.26	12.29	0.29	12.29	0.01
PC (16:0/18:0) $[M+Na]^+$	784.5827	22.21	22.69	2.14	22.70	0.03	12.73	12.78	0.40	12.79	0.08
			**MRE (%)**	**2.84**		**0.05**			**0.23**		**0.06**

Supplementary Table S14: Corrections of t_p and t_0 from a measurement at 30°C to map with a measurement at 22°C of of 8 lipids in the mixture solution containing Major Mix and lipid standards. The t_p and t_0 corrections in this table were executed based on the correction equations in Supplementary Table S10.

**Compound**	***m/z***	**t_p 30°C (ms)**	**t_p 22°C (ms)**	**Pre-correct RE (%)**	**t_p 30°C to 22°C (ms)**	**Post-correct RE (%) (ms)**	**t_0 30°C (ms)**	**t_0 22°C (ms)**	**Pre-correct RE (%)**	**t_0 30°C to 22°C (ms)**	**Post-correct RE (%)**
LPC(16:0) $[M+H]^+$	496.3398	12.43	12.83	3.08	12.83	0.02	11.92	11.94	0.18	11.93	0.06
LPS(17:1) $[M+H]^+$	510.2826	11.22	11.64	3.57	11.61	0.25	11.76	11.77	0.03	11.77	0.01
LPC(16:0) $[M+Na]^+$	518.3216	12.96	13.36	2.98	13.36	0.04	12.01	12.04	0.26	12.03	0.10
LPS(17:1) $[M+Na]^+$	532.2645	11.59	12.03	3.64	11.98	0.40	11.82	11.84	0.10	11.83	0.03
DG(18:0) $[M+Na]^+$	647.5584	17.80	18.38	3.16	18.25	0.70	12.37	12.41	0.31	12.41	0.02
PE(18:0/18:2) $[M+H]^+$	744.5538	19.97	20.48	2.47	20.45	0.17	12.49	12.53	0.35	12.53	0.04
PC(16:0/18:0) $[M+H]^+$	763.6080	21.76	22.23	2.13	22.25	0.08	12.67	12.72	0.35	12.73	0.11
PE(18:0/18:2) $[M+Na]^+$	766.5357	20.75	21.29	2.53	21.23	0.28	12.52	12.55	0.26	12.57	0.14
PG(16:0/18:1) $[M+Na]^+$	771.5146	20.90	21.42	2.41	21.38	0.16	12.53	12.58	0.38	12.58	0.03
			**MRE (%)**	**2.89**		**0.23**			**0.25**		**0.06**

Supplementary Table S15: (Replicate experiment) corrections of t_p and t_0 from a measurement at 30°C to map with a measurement at 22°C of 5 non-lipid molecules in the mixture solution containing Major Mix and lipid standards. The data was used to construct a correction curve, yielding a linear equation of y = 1.0273x + 0.1471for t_p and y = 1.0433x - 0.4873 for t_0. The post-correction errors shown in this table were calculated based on the self-correction using the two equations.

**Compound**	***m/z***	**t_p 30°C (ms)**	**t_p 22°C (ms)**	**Pre-correct RE (%)**	**t_p 30°C to 22°C (ms)**	**Post-correct RE (%) (ms)**	**t_0 30°C (ms)**	**t_0 22°C (ms)**	**Pre-correct RE (%)**	**t_0 30°C to 22°C (ms)**	**Post-correct RE (%)**
Sulfadimethoxine $[M+H]^+$	311.0809	4.98	5.28	5.73	5.26	0.37	11.19	11.20	0.01	11.19	0.03
Val-Tyr-Val $[M+H]^+$	380.2180	7.57	7.91	4.30	7.92	0.18	11.42	11.42	0.01	11.43	0.05
Terfenadine $[M+H]^+$	472.3210	11.71	12.14	3.58	12.18	0.27	11.81	11.84	0.22	11.84	0.02
Leucine Enkephalin $[M+H]^+$	556.2771	11.90	12.37	3.80	12.37	0.01	11.82	11.85	0.22	11.84	0.02
Reserpine $[M+H]^+$	609.2807	14.60	15.17	3.77	15.14	0.17	12.09	12.12	0.29	12.12	0.00
			**MRE (%)**	**4.24**		**0.20**			**0.15**		**0.02**

Supplementary Table S16: (Replicate experiment) corrections of t_p and t_0 from a measurement at 30°C to map with a measurement at 22°C of of 8 lipids in the mixture solution containing Major Mix and lipid standards. The t_p and t_0 corrections in this table were executed based on the correction equations in Supplementary Table S12.

Compound	*m/z*	t_p 30°C (ms)	t_p 22°C (ms)	Pre-correct RE (%)	t_p 30°C to 22°C (ms)	Post-correct RE (%) (ms)	t_0 30°C (ms)	t_0 22°C (ms)	Pre-correct RE (%)	t_0 30°C to 22°C (ms)	Post-correct RE (%)
LPE 16:0 $[M+H]^+$	454.2928	10.49	10.83	3.15	10.93	0.85	11.70	11.72	0.15	11.72	0.02
LPE 16:0 $[M+Na]^+$	476.2747	11.26	11.61	3.07	11.71	0.84	11.79	11.81	0.21	11.81	0.02
LPC 16:0 $[M+H]^+$	496.3398	12.42	12.80	2.92	12.91	0.88	11.95	11.97	0.20	11.98	0.05
LPS 17:1 $[M+H]^+$	510.2826	11.21	11.60	3.31	11.67	0.60	11.76	11.79	0.20	11.78	0.01
LPC 16:0 $[M+Na]^+$	518.3216	12.95	13.32	2.73	13.45	1.03	12.01	12.04	0.24	12.05	0.03
LPS 17:1 $[M+Na]^+$	532.2645	11.58	11.98	3.37	12.04	0.50	11.83	11.85	0.20	11.85	0.01
Cer (d18:1/17:0) $[M+H]^+$	552.5350	16.45	16.91	2.67	17.05	0.85	12.30	12.34	0.36	12.34	0.00
Cer (d18:1/17:0) $[M+Na]^+$	574.5169	16.02	16.41	2.37	16.61	1.19	12.26	12.29	0.26	12.31	0.10
DG 18:0 $[M+Na]^+$	647.5584	17.88	18.30	2.32	18.51	1.15	12.37	12.41	0.28	12.42	0.11
PE (18:0/18:2) $[M+H]^+$	744.5538	19.97	20.41	2.14	20.66	1.25	12.49	12.53	0.28	12.55	0.14
PC (16:0/18:0) $[M+H]^+$	763.6080	21.75	22.20	2.04	22.49	1.30	12.68	12.72	0.32	12.74	0.16
PE (18:0/18:2) $[M+Na]^+$	766.5357	20.74	21.26	2.46	21.45	0.89	12.52	12.55	0.24	12.58	0.20

PG 16:0/18:1 [M+Na] +	771.5146	20.87	21.34	2.18	21.59	1.18	12.54	12.58	0.31	12.60	0.13
PC (16:0/18:0) [M+Na] +	784.5827	22.14	22.62	2.13	22.89	1.20	12.72	12.77	0.38	12.79	0.12
			**MRE (%)**	**2.63**		**0.98**			**0.26**		**0.08**

Supplementary Table S17: (Replicate experiment) corrections of t_p and t_0 from a measurement at 30°C to map with a measurement at 22°C of 5 lipid molecules in the mixture solution containing Major Mix and lipid standards. The lipids were selected based on them having the lowest, medium, and highest drift time to cover the entire drift time range of this experiment. The data was used to construct a correction curve, yielding a linear equation of $y = 1.0121x + 0.2192$ for t_p and $y = 1.0297x - 0.3275$ for t_0. The post-correction errors shown in this table were calculated based on the self-correction using the two equations.

**Compound**	***m/z***	**t_p 30°C (ms)**	**t_p 22°C (ms)**	**Pre-correct RE (%)**	**t_p 30°C to 22°C (ms)**	**Post-correct RE (%) (ms)**	**t_0 30°C (ms)**	**t_0 22°C (ms)**	**Pre-correct RE (%)**	**t_0 30°C to 22°C (ms)**	**Post-correct RE (%)**
LPE(16:0) $[M+H]^+$	454.2928	10.49	10.83	3.15	10.84	0.05	11.70	11.72	0.15	11.72	0.03
LPE(16:0) $[M+Na]^+$	476.2747	11.26	11.61	3.07	11.61	0.01	11.79	11.81	0.21	11.81	0.02
Cer(d18:1/17:0) $[M+H]^+$	552.5350	16.45	16.91	2.67	16.87	0.20	12.30	12.34	0.36	12.34	0.06
Cer(d18:1/17:0) $[M+Na]^+$	574.5169	16.02	16.41	2.37	16.44	0.15	12.26	12.29	0.26	12.30	0.04
PC (16:0/18:0) $[M+Na]^+$	784.5827	22.14	22.62	2.13	22.62	0.03	12.72	12.77	0.38	12.77	0.01
			**MRE (%)**	**2.68**		**0.09**			**0.27**		**0.03**

Supplementary Table S18: (Replicate experiment) corrections of t_p and t_0 from a measurement at 30°C to map with a measurement at 22°C of of 8 lipids in the mixture solution containing Major Mix and lipid standards. The t_p and t_0 corrections in this table were executed based on the correction equations in Supplementary Table S14.

**Compound**	***m/z***	**t_p 30°C (ms)**	**t_p 22°C (ms)**	**Pre-correct RE (%)**	**t_p 30°C to 22°C (ms)**	**Post-correct RE (%) (ms)**	**t_0 30°C (ms)**	**t_0 22°C (ms)**	**Pre-correct RE (%)**	**t_0 30°C to 22°C (ms)**	**Post-correct RE (%)**
LPC(16:0) $[M+H]^+$	496.3398	12.42	12.80	2.92	12.79	0.03	11.95	11.97	0.20	11.98	0.03
LPS(17:1) $[M+H]^+$	510.2826	11.21	11.60	3.31	11.57	0.25	11.76	11.79	0.20	11.78	0.02
LPC(16:0) $[M+Na]^+$	518.3216	12.95	13.32	2.73	13.33	0.09	12.01	12.04	0.24	12.04	0.00
LPS(17:1) $[M+Na]^+$	532.2645	11.58	11.98	3.37	11.94	0.37	11.83	11.85	0.20	11.85	0.00
DG(18:0) $[M+Na]^+$	647.5584	17.88	18.30	2.32	18.31	0.06	12.37	12.41	0.28	12.41	0.05
PE(18:0/18:2) $[M+H]^+$	744.5538	19.97	20.41	2.14	20.43	0.12	12.49	12.53	0.28	12.54	0.06
PC(16:0/18:0) $[M+H]^+$	763.6080	21.75	22.20	2.04	22.23	0.14	12.68	12.72	0.32	12.73	0.06
PE(18:0/18:2) $[M+Na]^+$	766.5357	20.74	21.26	2.46	21.21	0.25	12.52	12.55	0.24	12.57	0.11
PG(16:0/18:1) $[M+Na]^+$	771.5146	20.87	21.34	2.18	21.34	0.03	12.54	12.58	0.31	12.59	0.04
			**MRE (%)**	**2.61**		**0.15**			**0.25**		**0.04**

Supplementary Table 19: Reconstructed multi-pass arrival time database of lipid standards from passes 1 to 10 in ESI+. Values highlighted in green represent the arrival times (t_n) of the calibrant lipids measured from our DESI-cIM-MS experiment. Values highlighted in yellow correspond to the arrival times that best match the corrected measured tn of the unknown compounds from the experiment.

Name	Adduct	*m/z*	t_p (ms)	t_0 (ms)	t_n (ms)									
					1	2	3	4	5	6	7	8	9	10
LPE(16:0)	$[M+H]^+$	454.2928	10.83	11.72	22.55	33.39	44.22	55.05	65.89	76.72	87.55	98.39	109.22	120.06
LPC(13:0)	$[M+H]^+$	454.2928	11.40	11.76	23.16	34.55	45.95	57.34	68.74	80.14	91.53	102.93	114.32	125.72
LPE(16:0)	$[M+Na]^+$	476.2747	11.62	11.81	23.43	35.04	46.66	58.28	69.89	81.51	93.12	104.74	116.35	127.97
LPC(13:0)	$[M+Na]^+$	476.2748	12.07	11.88	23.95	36.02	48.09	60.17	72.24	84.31	96.38	108.45	120.53	132.60
LPC(16:0)	$[M+H]^+$	496.3398	12.80	11.97	24.77	37.57	50.37	63.16	75.96	88.76	101.56	114.36	127.15	139.95
LPS(17:1)	$[M+H]^+$	510.2826	11.60	11.79	23.38	34.98	46.58	58.18	69.78	81.37	92.97	104.57	116.17	127.77
LPC(16:0)	$[M+Na]^+$	518.3216	13.31	12.04	25.35	38.66	51.97	65.29	78.60	91.91	105.22	118.53	131.84	145.15
LPS(17:1)	$[M+Na]^+$	532.2645	11.98	11.85	23.83	35.80	47.78	59.75	71.73	83.71	95.68	107.66	119.64	131.61
Cer(d18:1/17:0)	$[M+H-H2O]^+$	534.5244	16.64	12.31	28.95	45.58	62.22	78.86	95.50	112.13	128.77	145.41	162.05	178.69
Cer(d18:1/17:0)	$[M+H]^+$	552.535	16.91	12.34	29.25	46.15	63.06	79.96	96.87	113.78	130.68	147.59	164.49	181.40
Cer(d18:1/17:0)	$[M+Na]^+$	574.5169	16.41	12.29	28.70	45.11	61.52	77.93	94.34	110.75	127.16	143.57	159.97	176.38
DG(18:0)	$[M+Na]^+$	647.5584	18.30	12.41	30.71	49.01	67.31	85.61	103.91	122.21	140.51	158.81	177.11	195.42
PE(18:0/18:2)	$[M+H]^+$	744.5538	20.41	12.53	32.94	53.34	73.75	94.16	114.57	134.97	155.38	175.79	196.20	216.60
PC (16:0/18:0)	$[M+H]^+$	762.6007	22.18	12.72	34.90	57.08	79.26	101.44	123.63	145.81	167.99	190.17	212.35	234.54
PE(18:0/18:2)	$[M+Na]^+$	766.5357	21.26	12.55	33.81	55.07	76.33	97.59	118.85	140.11	161.37	182.63	203.89	225.15
PG(16:0/18:1)	$[M+Na]^+$	771.5146	21.34	12.58	33.92	55.25	76.59	97.92	119.26	140.60	161.93	183.27	204.60	225.94
PC (16:0/18:0)	$[M+Na]^+$	784.5827	22.59	12.77	35.36	57.95	80.54	103.14	125.73	148.32	170.91	193.50	216.09	238.68

Supplementary Table 20: Measured values and database values of multi-pass arrival times of the calibrant lipids in our DESI-cIM-MS. The data was used to construct an arrival time correction line, yielding a linear equation of $y = 0.9521x + 2.9161$ ($R^2 = 0.9978$). This equation was then used to correct t_n of the unknown compounds in this measurement. Room temperature was at 20.5°C at the beginning of the measurement.

**Name**	***m/z***	**Adduct**	**Measured t_n (ms)**	**Database t_n (ms)**
LPC(16:0)	496.3433	$[M+H]^+$	117.33	114.36
LPC(16:0)	518.3256	$[M+Na]^+$	121.49	118.53
Cer(d18:1/17:0)	552.5387	$[M+H]^+$	116.31	113.78
Cer(d18:1/17:0)	574.5257	$[M+Na]^+$	113.10	110.75
PC (16:0/18:0)	762.6052	$[M+H]^+$	127.15	123.63
PC (16:0/18:0)	784.5886	$[M+Na]^+$	128.58	125.73

Supplementary Table S21: Corrections of t_p and t_0 of the small molecules in the Major Mix solution measured with a machine in Singapore (SG) to map those measured with a machine in Thailand (TH). The data was used to construct a correction curve, yielding a linear equation of y = 1.0249x + 0.0633 for t_p and y = 0.9880x + 0.1119 for t_0. The post-correction errors shown in this table were calculated based on the self-correction using the two equations.

**Compound**	***m/z***	**t_p SG (ms)**	**t_p TH (ms)**	**Pre-correct RE (%)**	**t_p SG to TH (ms)**	**Post-correct RE (%) (ms)**	**t_0 SG (ms)**	**t_0 TH (ms)**	**Pre-correct RE (%)**	**t_0 SG to TH (ms)**	**Post-correct RE (%)**
Sulfadimethoxine $[M+H]^+$	311.0824	5.12	5.31	3.54	5.32	0.27	11.30	11.29	0.16	11.28	0.05
Sulfadimethoxine $[M+Na]^+$	333.0647	5.75	5.97	3.67	5.97	0.03	11.37	11.35	0.17	11.35	0.04
Val-Tyr-Val $[M+H]^+$	380.2198	7.66	7.91	3.11	7.92	0.23	11.61	11.58	0.30	11.59	0.06
Terfenadine $[M+H]^+$	472.3220	11.84	12.19	2.88	12.21	0.13	12.06	12.02	0.31	12.02	0.03
Leucine Enkephalin $[M+H]^+$	556.2772	12.05	12.41	2.94	12.42	0.06	12.05	12.01	0.31	12.02	0.04
Leucine Enkephalin $[M+Na]^+$	578.2571	12.25	12.62	2.87	12.63	0.13	12.08	12.04	0.35	12.05	0.08
Reserpine $[M+H]^+$	609.2821	14.79	15.24	2.90	15.23	0.01	12.26	12.24	0.14	12.22	0.15
			**MRE (%)**	**3.13**		**0.12**			**0.25**		**0.06**

Supplementary Table S22: Lipid calibrants using to calibrate t_p from a human serum measurement at 30°C to map with a human serum measurement at 20°C. The data was used to construct a correction curve, yielding a linear equation of y = 1.0277x + 0.2342. The post-correction REs shown in this table were calculated based on the self-correction.

**Compound**	***m/z***	**t_p 30°C (ms)**	**t_p 20°C (ms)**	**Pre-correct RE (%)**	**t_p 30°C to 20°C (ms)**	**Post-correct RE (%) (ms)**
LPC(16:0) $[M+H]^+$	496.3431	12.43	13.02	4.76	13.01	0.11
LPC(16:0) $[M+Na]^+$	518.3233	12.93	13.51	4.45	13.53	0.12
LPE(16:0) $[M+H]^+$	454.2948	10.49	11.02	5.04	11.02	0.04
LPE(16:0) $[M+Na]^+$	476.2759	11.25	11.80	4.84	11.80	0.01
PE(16:0/18:1) $[M+H]^+$	718.5406	19.26	19.99	3.83	20.02	0.15
PE(16:0/18:1) $[M+Na]^+$	740.5233	20.17	20.99	4.07	20.96	0.13
			**MRE (%)**	**4.50**		**0.09**

Supplementary Table S23: Lipid calibrants using to calibrate tp from a mouse serum measurement at 30°C to map with a mouse serum measurement at 20°C. The data was used to construct a correction curve, yielding a linear equation of y = 1.0287x + 0.2610. The post-correction errors shown in this table were calculated based on the self-correction.

**Compound**	***m/z***	**t_p 30°C (ms)**	**t_p 20°C (ms)**	**Pre-correct RE (%)**	**t_p 30°C to 20°C (ms)**	**Post-correct RE (%) (ms)**
LPC(16:0) $[M+H]^+$	496.3421	12.40	13.04	4.88	13.02	0.14
LPC(16:0) $[M+Na]^+$	518.3250	12.89	13.50	4.56	13.52	0.11
LPE(16:0) $[M+H]^+$	454.2945	10.45	11.02	5.14	11.01	0.05
LPE(16:0) $[M+Na]^+$	476.2769	11.22	11.79	4.88	11.80	0.07
PE(16:0/18:1) $[M+H]^+$	718.5405	19.20	19.99	3.92	20.01	0.14
PE(16:0/18:1) $[M+Na]^+$	740.5230	20.11	20.98	4.12	20.95	0.12
			**MRE (%)**	**4.58**		**0.11**

Supplementary Table S24: Corrections from a human serum measurement at 30°C to map with a human serum measurement at 20°C s based on the correction equations in Supplementary Table S17. The features with post-correction error greater than 0.7% are highlighted in yellow. Feature associated to leucine enkephalin, the only non-lipid chemical standard added to the serum, are made italic.

Peak No.	*m/z*	t_p 30°C (ms)	t_p 20°C (ms)	Pre-correct RE (%)	t_p 30°C to 20°C (ms)	Post-correct RE (%) (ms)
1	496.3431	12.43	13.02	4.55	13.01	0.11
2	518.3233	12.93	13.51	4.26	13.53	0.12
3	758.5734	20.73	21.51	3.61	21.54	0.15
4	454.2948	10.49	11.02	4.80	11.02	0.04
5	497.3447	12.44	13.03	4.56	13.02	0.12
6	786.6045	21.78	22.56	3.44	22.62	0.27
7	760.5869	21.19	21.98	3.62	22.01	0.11
8	782.5714	21.22	22.09	3.94	22.04	0.22
9	524.3718	13.44	14.07	4.49	14.05	0.18
10	663.4549	18.62	19.57	4.84	19.37	1.01
11	685.4369	19.25	20.25	4.93	20.02	1.14
12	780.5552	21.15	22.01	3.90	21.97	0.18
13	718.5406	19.26	19.99	3.69	20.02	0.15
14	***556.2772***	***11.89***	***12.60***	***5.65***	***12.45***	***1.17***
15	476.2759	11.25	11.80	4.61	11.80	0.01
16	784.5871	21.43	22.30	3.89	22.26	0.18
17	703.5748	20.55	21.31	3.57	21.36	0.20
18	519.3257	12.94	13.51	4.26	13.53	0.13
19	808.5869	22.22	23.13	3.92	23.07	0.25
20	520.3389	12.13	12.72	4.65	12.70	0.17
21	810.6032	22.31	23.18	3.77	23.16	0.09
22	783.5757	21.23	22.05	3.72	22.05	0.01

23	664.4581	18.63	19.58	4.87	19.38	1.04
24	546.3534	13.85	14.46	4.25	14.46	0.02
25	455.2972	10.49	11.02	4.76	11.02	0.00
26	522.3555	12.83	13.43	4.53	13.42	0.14
27	686.4401	19.26	20.24	4.84	20.03	1.05
28	781.5591	21.15	22.02	3.95	21.97	0.23
29	788.6164	22.12	22.82	3.04	22.97	0.67
30	806.5702	21.80	22.67	3.83	22.64	0.13
31	785.5936	21.44	22.29	3.79	22.27	0.07
32	774.5693	21.12	21.94	3.75	21.94	0.01
33	557.2801	11.89	12.60	5.65	12.45	1.18
34	740.5233	20.17	20.99	3.91	20.96	0.13
35	577.5196	16.74	17.43	3.95	17.44	0.05
36	478.3292	11.96	12.54	4.58	12.53	0.07
37	440.4112	11.55	12.13	4.81	12.10	0.24
38	809.5915	22.23	23.11	3.81	23.08	0.13
39	798.5669	21.58	22.47	3.95	22.42	0.25
40	796.5507	21.50	22.42	4.08	22.33	0.38
41	498.3472	12.44	13.04	4.58	13.02	0.14
42	804.5572	21.76	22.63	3.84	22.60	0.14
43	436.2837	10.47	11.02	4.95	10.99	0.19
44	813.684	24.06	24.97	3.64	24.96	0.03
45	812.6205	22.54	23.40	3.67	23.40	0.01
46	477.2797	11.25	11.80	4.67	11.80	0.05
47	756.5562	20.41	21.26	3.98	21.21	0.22
48	811.6109	22.37	23.23	3.72	23.22	0.04
49	542.3226	12.52	13.18	5.07	13.10	0.67
50	671.5749	20.89	21.95	4.81	21.71	1.11
51	800.5824	21.80	22.69	3.92	22.64	0.23
52	762.5956	21.15	21.98	3.77	21.97	0.04
53	725.5576	20.68	21.45	3.59	21.49	0.17

54	824.5805	22.43	23.39	4.09	23.28	0.43
55	881.7585	27.03	28.03	3.59	28.01	0.08
56	822.5657	21.91	22.97	4.62	22.75	0.96
57	802.5959	22.13	23.02	3.87	22.97	0.19
58	687.568	21.36	22.28	4.13	22.19	0.42
59	547.3571	13.86	14.47	4.23	14.47	0.04
60	807.5742	21.79	22.71	4.08	22.62	0.39
61	521.3427	12.13	12.73	4.65	12.70	0.17
62	879.7446	26.57	27.57	3.65	27.54	0.13
63	459.2496	10.34	10.86	4.76	10.86	0.04
64	741.5276	20.18	20.99	3.87	20.97	0.10
65	826.5971	22.75	23.62	3.70	23.61	0.04
66	775.5722	21.13	21.94	3.71	21.95	0.03
67	832.5854	22.84	23.75	3.81	23.71	0.16
68	820.552	21.92	22.86	4.11	22.76	0.43
69	687.4434	19.25	20.28	5.10	20.01	1.31
70	720.5496	19.33	20.00	3.37	20.10	0.48
71	797.5534	21.48	22.34	3.85	22.31	0.14
72	789.6205	22.24	23.05	3.48	23.09	0.21
73	805.5592	21.78	22.64	3.78	22.62	0.08
74	815.7003	24.55	25.48	3.67	25.46	0.08
75	814.6872	24.06	24.98	3.70	24.96	0.10
76	731.6065	21.49	22.31	3.66	22.32	0.06
77	497.5946	12.43	13.03	4.57	13.01	0.13
78	660.2911	19.08	20.13	5.23	19.84	1.44
79	834.602	22.86	23.79	3.94	23.72	0.30
80	534.2969	13.08	13.67	4.31	13.68	0.06
81	701.5569	20.01	20.82	3.85	20.80	0.06
82	705.5823	20.55	21.32	3.63	21.35	0.14
			**MRE (%)**	**4.15**		**0.28**

Supplementary Table S25: Corrections from a mouse serum measurement at 30°C to map with a mouse serum measurement at 20°C s based on the correction equations in Supplementary Table S18. The compounds with post-correction error greater than 0.7% are highlighted in yellow. Feature associated to leucine enkephalin, the only non-lipid chemical standard added to the serum, are made italic.

**Peak No.**	***m/z***	**t_p 30°C (ms)**	**t_p 20°C (ms)**	**Pre-correct RE (%)**	**t_p 30°C to 20°C (ms)**	**Post-correct RE (%) (ms)**
1	518.325	12.89	13.50	4.56	13.52	0.11
2	685.4388	19.17	20.20	5.12	19.98	1.11
3	496.3421	12.40	13.04	4.88	13.02	0.14
4	476.2769	11.22	11.79	4.88	11.80	0.07
5	546.3545	13.81	14.46	4.46	14.47	0.09
6	454.2945	10.45	11.02	5.14	11.01	0.05
7	686.4407	19.17	20.21	5.14	19.98	1.12
8	519.3263	12.90	13.51	4.52	13.53	0.15
9	***556.2774***	***11.84***	***12.59***	***5.94***	***12.44***	***1.17***
10	524.372	13.40	14.08	4.81	14.05	0.23
11	740.523	20.11	20.98	4.12	20.95	0.12
12	718.5405	19.20	19.99	3.92	20.01	0.14
13	497.3443	12.41	13.04	4.85	13.02	0.12
14	663.4552	18.55	19.57	5.22	19.34	1.17
15	782.5703	21.53	22.42	4.01	22.40	0.09
16	477.2795	11.22	11.80	4.88	11.80	0.07
17	547.3571	13.82	14.47	4.49	14.47	0.05
18	711.57	21.52	22.66	5.06	22.40	1.18
19	881.7604	26.94	28.00	3.80	27.97	0.11
20	741.5273	20.10	20.97	4.17	20.93	0.18
21	808.5872	22.18	23.12	4.04	23.08	0.16
22	671.5751	20.85	21.97	5.11	21.71	1.20
23	455.2974	10.46	11.03	5.19	11.02	0.10

24	879.7451	26.53	27.59	3.83	27.55	0.12
25	577.5195	16.70	17.43	4.19	17.44	0.05
26	687.5691	21.26	22.36	4.90	22.14	1.00
27	760.5866	21.17	22.02	3.89	22.03	0.06
28	440.4114	11.51	12.13	5.09	12.10	0.22
29	780.555	21.12	22.03	4.12	21.99	0.19
30	687.4429	19.18	20.21	5.11	19.99	1.10
31	557.2804	11.85	12.60	5.95	12.45	1.18
32	459.2496	10.30	10.86	5.11	10.86	0.02
33	703.5703	20.53	21.31	3.69	21.38	0.30
34	786.6042	21.69	22.59	4.01	22.57	0.10
35	664.4588	18.57	19.59	5.21	19.36	1.16
36	783.5732	21.55	22.43	3.91	22.43	0.02
37	660.2919	18.99	20.05	5.29	19.80	1.27
38	712.5735	21.53	22.67	5.03	22.41	1.15
39	725.557	20.67	21.47	3.71	21.53	0.27
40	436.2839	10.44	11.01	5.15	11.00	0.06
41	758.5718	20.73	21.56	3.88	21.58	0.09
42	810.6021	22.48	23.50	4.32	23.39	0.46
43	673.5886	20.87	21.96	4.96	21.73	1.04
44	498.2581	11.46	12.01	4.63	12.05	0.28
45	809.5919	22.17	23.10	4.01	23.07	0.13
46	695.5752	21.33	22.49	5.13	22.21	1.25
47	743.5581	22.63	23.42	3.37	23.54	0.52
48	853.7295	25.72	26.86	4.22	26.72	0.49
49	728.5682	21.61	22.80	5.22	22.49	1.35
50	761.5897	21.21	22.02	3.69	22.08	0.26
51	685.5576	21.30	22.21	4.10	22.17	0.17
52	568.4578	15.33	16.04	4.40	16.03	0.03
53	495.2995	12.44	13.21	5.82	13.06	1.14
54	784.5824	21.52	22.39	3.87	22.40	0.05

55	877.7299	26.06	27.11	3.85	27.07	0.13
56	701.4119	19.43	20.47	5.10	20.25	1.10
57	781.5583	21.12	22.04	4.16	21.99	0.23
58	579.2626	12.06	12.81	5.81	12.67	1.07
59	544.3382	13.03	13.69	4.85	13.66	0.22
60	504.3429	12.44	13.08	4.91	13.05	0.18
61	704.5731	20.46	21.28	3.86	21.31	0.12
62	534.2965	13.05	13.66	4.47	13.69	0.18
63	905.7591	27.12	28.26	4.05	28.16	0.37
64	742.5293	20.14	21.01	4.14	20.98	0.14
65	855.7437	26.01	27.08	3.95	27.02	0.23
66	445.3157	9.51	10.12	6.02	10.05	0.74
67	788.617	22.22	23.05	3.60	23.12	0.30
68	530.3593	12.79	13.46	4.96	13.42	0.29
69	907.772	27.58	28.66	3.77	28.64	0.10
70	720.5496	19.20	19.99	3.93	20.01	0.14
71	541.4296	14.36	15.03	4.44	15.04	0.04
72	507.2736	11.95	12.72	6.01	12.56	1.25
73	696.5785	21.36	22.49	5.01	22.24	1.13
74	560.3721	14.30	14.96	4.42	14.97	0.06
75	813.6856	24.02	24.99	3.90	24.97	0.10
76	883.7683	26.96	28.04	3.88	27.99	0.19
			**MRE (%)**	**4.57**		**0.43**

Supplementary Table S26: Self-correction of t_p of the spiked lipid standards from a mouse serum (M) measurement at 30°C to map with a human serum (H) measurement at 20°C. The correction equation was y = 1.0300x + 0.2447. The post-correction errors shown in this table were calculated based on the self-correction.

**Compound**	***m/z***	**t_p M 30°C (ms)**	**t_p H 20°C (ms)**	**Pre-correct RE (%)**	**t_p M 30°C to H 20°C (ms)**	**Post-correct RE (%) (ms)**
LPC(16:0) $[M+H]^+$	496.3421	12.40	13.02	4.76	13.02	0.03
LPC(16:0) $[M+Na]^+$	518.3250	12.89	13.51	4.59	13.52	0.08
LPE(16:0) $[M+H]^+$	454.2945	10.45	11.02	5.17	11.01	0.11
LPE(16:0) $[M+Na]^+$	476.2769	11.22	11.80	4.92	11.80	0.01
PE(16:0/18:1) $[M+H]^+$	718.5405	19.20	19.99	3.95	20.02	0.15
PE(16:0/18:1) $[M+Na]^+$	740.5230	20.11	20.99	4.19	20.96	0.15
			**MRE (%)**	**4.60**		**0.09**

Supplementary Table S27: Lipid calibrants using to calibrate t_p from a human serum measurement at 20°C with a traveling wave height (TWH) of 15V to those with the TWH of 22V. The data was used to construct a correction curve, yielding a linear equation of y = 0.4859x - 0.9481. The post-correction errors shown in this table were calculated based on the self-correction.

**Compound**	***m/z***	**t_p 15 V TWH (ms)**	**t_p 22 V TWH (ms)**	**Pre-correct RE (%)**	**t_p 15 V to 22 V (ms)**	**Post-correct RE (%) (ms)**
LPC(16:0) [M+H]+	496.3431	28.77	13.02	120.93	13.03	0.07
LPC(16:0) [M+Na]+	518.3233	29.70	13.51	119.84	13.48	0.20
LPE(16:0) [M+H]+	454.2948	24.64	11.02	123.63	11.03	0.06
LPE(16:0) [M+Na]+	476.2759	26.23	11.80	122.38	11.80	0.02
PE(16:0/18:1) [M+H]+	718.5406	43.19	19.99	116.01	20.04	0.22
PE(16:0/18:1) [M+Na]+	740.5233	45.08	20.99	114.76	20.96	0.17
			**MRE (%)**	**119.59**		**0.12**

Supplementary Table S28: Corrections from a human serum measurement at 20°C with a traveling wave height (TWH) of 15V to map with those with the TWH of 22V based on the correction equations in Supplementary Table S22. The compounds with post-correction error greater than 0.7% are highlighted in yellow. Feature associated to leucine enkephalin, the only non-lipid chemical standard added to the serum, are made italic. The two features that exhibited post-correction errors when using non-lipid features as calibrants than when using lipid standards as calibrants are highlighted in red.

**Peak No.**	***m/z***	**t_p 15 V TWH (ms)**	**t_p 22 V TWH (ms)**	**Pre-correct RE (%)**	**t_p 15 V to 22 V (ms)**	**Post-correct RE (%) (ms)**
1	496.3431	28.77	13.02	120.93	13.03	0.07
2	518.3233	29.70	13.51	119.84	13.48	0.20
3	758.5734	46.13	21.51	114.47	21.46	0.20
4	454.2948	24.64	11.02	123.63	11.03	0.06
5	497.3447	28.78	13.03	120.81	13.03	0.02
6	786.6045	48.40	22.56	114.59	22.57	0.07
7	760.5869	47.07	21.98	114.13	21.92	0.27
8	782.5714	47.41	22.09	114.57	22.09	0.03
9	524.3718	31.08	14.07	120.79	14.15	0.55
10	663.4549	42.68	19.57	118.11	19.79	1.13
11	685.4369	44.15	20.25	118.04	20.50	1.26
12	780.5552	47.20	22.01	114.49	21.99	0.09
13	718.5406	43.19	19.99	116.01	20.04	0.22
14	556.2772	28.32	12.60	124.83	12.81	1.72
15	476.2759	26.23	11.80	122.38	11.80	0.02
16	784.5871	47.67	22.30	113.75	22.21	0.39
17	703.5748	45.63	21.31	114.08	21.22	0.43
18	519.3257	29.71	13.51	119.84	13.49	0.20
19	520.3389	28.19	12.72	121.59	12.75	0.22
20	783.5757	47.41	22.05	115.02	22.09	0.18
21	664.4581	42.68	19.58	117.97	19.79	1.07
22	546.3534	31.77	14.46	119.72	14.49	0.21

23	455.2972	24.65	11.02	123.74	11.03	0.11
24	522.3555	29.66	13.43	120.80	13.47	0.23
25	686.4401	44.14	20.24	118.09	20.50	1.29
26	781.5591	47.20	22.02	114.33	21.99	0.16
27	788.6164	48.73	22.82	113.55	22.73	0.39
28	806.5702	48.55	22.67	114.16	22.64	0.12
29	785.5936	47.67	22.29	113.91	22.22	0.32
30	774.5693	47.00	21.94	114.18	21.89	0.25
31	557.2801	28.33	12.60	124.77	12.82	1.69
32	740.5233	45.08	20.99	114.76	20.96	0.17
33	577.5196	37.85	17.43	117.18	17.45	0.09
34	440.4112	27.04	12.13	122.94	12.19	0.51
35	798.5669	48.08	22.47	113.97	22.41	0.25
36	796.5507	47.70	22.42	112.80	22.23	0.83
37	498.3472	28.79	13.04	120.84	13.04	0.03
38	804.5572	48.48	22.63	114.19	22.61	0.11
39	436.2837	24.66	11.02	123.83	11.03	0.15
40	477.2797	26.24	11.80	122.32	11.80	0.01
41	756.5562	45.36	21.26	113.41	21.09	0.76
42	542.3226	29.38	13.18	122.83	13.33	1.08
43	671.5749	47.59	21.95	116.83	22.18	1.04
44	800.5824	48.66	22.69	114.47	22.70	0.03
45	762.5956	47.11	21.98	114.31	21.94	0.18
46	725.5576	45.93	21.45	114.08	21.37	0.40
47	802.5959	49.26	23.02	114.05	22.99	0.11
48	687.568	48.24	22.28	116.46	22.49	0.92
49	547.3571	31.77	14.47	119.58	14.49	0.14
50	807.5742	48.56	22.71	113.78	22.65	0.30
51	521.3427	28.21	12.73	121.66	12.76	0.26
52	459.2496	24.27	10.86	123.58	10.85	0.09
53	741.5276	45.08	20.99	114.74	20.96	0.17

54	775.5722	47.01	21.94	114.24	21.89	0.22
55	820.552	48.93	22.86	114.06	22.83	0.14
56	681.4207	42.79	19.64	117.80	19.84	1.00
57	687.4434	44.19	20.28	117.90	20.52	1.20
58	720.5496	43.14	20.00	115.66	20.01	0.05
59	797.5534	47.87	22.34	114.23	22.31	0.15
60	789.6205	49.46	23.05	114.62	23.09	0.17
61	805.5592	48.45	22.64	114.02	22.59	0.19
62	731.6065	47.68	22.31	113.76	22.22	0.38
63	497.5946	28.77	13.03	120.81	13.03	0.01
64	660.2911	44.13	20.13	119.21	20.50	1.80
65	534.2969	30.08	13.67	120.00	13.67	0.04
66	701.5569	44.78	20.82	115.13	20.81	0.02
67	705.5823	45.67	21.32	114.19	21.24	0.37
68	790.5676	47.82	22.36	113.87	22.29	0.32
69	799.5698	48.07	22.51	113.51	22.41	0.47
70	814.5653	48.93	22.81	114.54	22.83	0.09
71	496.5844	28.77	13.03	120.87	13.03	0.04
72	768.5885	47.77	22.34	113.82	22.26	0.35
73	482.3575	29.08	13.17	120.74	13.18	0.06
74	433.2344	22.66	10.05	125.53	10.06	0.15
75	801.5853	48.70	22.74	114.13	22.72	0.12
76	734.5707	46.09	21.55	113.90	21.45	0.47
			**MRE (%)**	**117.32**		**0.38**
